# Body mass index, abdominal fatness, fat mass and the risk of atrial fibrillation: a systematic review and dose–response meta-analysis of prospective studies

**DOI:** 10.1007/s10654-017-0232-4

**Published:** 2017-02-13

**Authors:** Dagfinn Aune, Abhijit Sen, Sabrina Schlesinger, Teresa Norat, Imre Janszky, Pål Romundstad, Serena Tonstad, Elio Riboli, Lars J. Vatten

**Affiliations:** 1 Department of Public Health and General Practice, Faculty of Medicine, Norwegian University of Science and Technology, Trondheim, Norway; 2 Department of Epidemiology and Biostatistics, School of Public Health, Imperial College London, St. Mary’s Campus, Norfolk Place, Paddington, London, W2 1PG UK; 3 Bjørknes University College, Oslo, Norway; 4 Department of Preventive Cardiology, Oslo University Hospital Ullevål, Oslo, Norway

**Keywords:** Obesity, BMI, Waist circumference, Hip circumference, Waist-to-hip ratio, Fat mass, Fat percentage, Atrial fibrillation, Meta-analysis

## Abstract

Different adiposity measures have been associated with increased risk of atrial fibrillation, however, results have previously only been summarized for BMI. We therefore conducted a systematic review and meta-analysis of prospective studies to clarify the association between different adiposity measures and risk of atrial fibrillation. PubMed and Embase databases were searched up to October 24th 2016. Summary relative risks (RRs) were calculated using random effects models. Twenty-nine unique prospective studies (32 publications) were included. Twenty-five studies (83,006 cases, 2,405,381 participants) were included in the analysis of BMI and atrial fibrillation. The summary RR was 1.28 (95% confidence interval: 1.20–1.38, I^2^ = 97%) per 5 unit increment in BMI, 1.18 (95% CI: 1.12–1.25, I^2^ = 73%, n = 5) and 1.32 (95% CI: 1.16–1.51, I^2^ = 91%, n = 3) per 10 cm increase in waist and hip circumference, respectively, 1.09 (95% CI: 1.02–1.16, I^2^ = 44%, n = 4) per 0.1 unit increase in waist-to-hip ratio, 1.09 (95% CI: 1.02–1.16, I^2^ = 94%, n = 4) per 5 kg increase in fat mass, 1.10 (95% CI: 0.92–1.33, I^2^ = 90%, n = 3) per 10% increase in fat percentage, 1.10 (95% CI: 1.08–1.13, I^2^ = 74%, n = 10) per 5 kg increase in weight, and 1.08 (95% CI: 0.97–1.19, I^2^ = 86%, n = 2) per 5% increase in weight gain. The association between BMI and atrial fibrillation was nonlinear, *p*_nonlinearity_ < 0.0001, with a stronger association at higher BMI levels, however, increased risk was observed even at a BMI of 22–24 compared to 20. In conclusion, general and abdominal adiposity and higher body fat mass increase the risk of atrial fibrillation.

**Electronic supplementary material:**

The online version of this article (doi:10.1007/s10654-017-0232-4) contains supplementary material, which is available to authorized users.

## Introduction

The prevalence of overweight and obesity has increased rapidly over the last decades in all areas of the world [1]. Overweight and obesity are important risk factors for a wide range of chronic diseases, including cardiovascular diseases, type 2 diabetes, gallbladder disease, total mortality and several types of cancer [2–11], and the current trends are a major challenge for public health both in terms of reduced quality of life and increased medical costs [12].

Atrial fibrillation is the most common arrhythmia diagnosed in clinical practice [13] and globally there was an estimated 5 million incident cases in 2010 [14], while the prevalence was estimated at 33 million in 2015 [15]. The prevalence of atrial fibrillation has been projected to increase 2.5-fold in the next 50 years, mainly due to an aging population, but also due to an increased incidence of the disease [13]. Patients with atrial fibrillation are at increased risk of cardiovascular diseases including ischemic heart disease, heart failure, sudden cardiac death, stroke, as well as chronic kidney disease and all cause mortality [16]. The economic costs due to atrial fibrillation in the US has been estimated at more than $6 billion annually [17]. Overweight and obesity have been associated with increased risk of atrial fibrillation in several studies [18, 19]. Some studies suggested a J-shaped dose–response relationship between BMI and atrial fibrillation [20, 21], however, other studies suggested a linear association [22–28]. In addition, it is not clear whether other measures of body fatness such as waist circumference [26, 29–32], hip circumference [30, 32, 33], waist-to-hip ratio [29, 30, 32, 33], fat mass [30–32, 34], or body fat percentage [30, 31, 34] are associated with risk of atrial fibrillation or if the association differs by geographic location or ethnicity. Although a meta-analysis from 2008 found that both overweight and obesity as measured by body mass index (BMI) was associated with increased risk of atrial fibrillation [35], at least 20 additional studies involving >78,000 atrial fibrillation cases and >2.2 million participants have been published since that meta-analysis [20, 21, 23–31, 33, 34, 36–42]. Given the large number of additional studies that have been published since the previous meta-analysis and the availability of data regarding other adiposity measures as well, we conducted a systematic review and dose–response meta-analysis of prospective studies that investigated the association between body mass index, waist circumference, waist-to-hip ratio, or other measures of adiposity (hip circumference, fat mass, weight, weight gain) and the risk of atrial fibrillation.

## Methods

### Search strategy and inclusion criteria

We searched the PubMed and Embase databases up to October 24th 2016 for eligible studies (DA, SS and AS). A list of the search terms used are provided in Supplementary Tables 1 and 2. We followed standard criteria (MOOSE Guidelines) for reporting meta-analyses [43]. In addition, we searched the reference lists of previous meta-analyses [2, 35, 44] and the reference lists of the relevant publications for further studies. Study quality was assessed using the Newcastle–Ottawa scale [45].

### Study selection

We included prospective and retrospective cohort studies and nested case–control studies of the association between adiposity measures (BMI, waist circumference, and waist-to-hip ratio, hip circumference, body fat mass, fat percentage, weight, weight gain) and risk of atrial fibrillation that were published in English. Studies in high-risk populations (patient populations), abstract only publications, grey literature and unpublished studies were excluded. Adjusted relative risk (RR) estimates (hazard ratios, risk ratios, or odds ratios) had to be available with the 95% confidence intervals (95% CIs) in the publication and for the dose–response analysis, a quantitative measure of adiposity and the total number of cases and person-years or non-cases for at least 3 categories of the adiposity variable or on a continuous scale had to be available in the publication. When multiple publications were available from the same study we used the study with the largest number of cases, but when data on different anthropometric measures were covered by different publications from the same study both were included, but each study was only included once in each analysis. A list of the excluded studies and exclusion reasons are found in the Supplementary Table 3.

### Data extraction

We extracted the following data from each study: The first author’s last name, publication year, country where the study was conducted, study period, sample size, number of cases/controls, exposure variable, exposure level, relative risks and 95% confidence intervals for the highest versus the lowest level of the exposure variable and variables adjusted for in the analysis. Data were extracted by one reviewer (DA) and checked for accuracy by a second reviewer (AS).

### Statistical analysis

We calculated summary RRs and 95% CIs for a 5 unit increment in BMI, 10 cm increment in waist and hip circumference, a 0.1 unit increment in waist-to-hip ratio, and a 5 kg increment in fat mass and weight, 10% increase in body fat percentage, and 5% increase in weight gain using a random effects model [46]. For the primary analysis we used the model from each study that had the greatest degree of control for potential confounding with the exception of when potential intermediate risk factors were adjusted for in a separate step (as an exploration of how much of the association might be mediated by cholesterol for example). The average of the natural logarithm of the RRs was estimated and the RR from each study was weighted according to the method of DerSimonian and Laird [46]. A two-tailed *p* < 0.05 was considered statistically significant. If studies reported results separately for men and women or other subgroups we combined the subgroup-specific estimates using a fixed-effects model to generate an overall estimate so that each study was only represented once in the main analysis, but sex-specific results are presented separately in subgroup analyses.

The method described by Greenland and Longnecker [47] was used for the dose–response analysis and we calculated study-specific slopes (linear trends) and 95% CIs from the natural logs of the reported RRs and CIs across categories of each adiposity measure. The mean or median level of each adiposity measure in each category was assigned to the corresponding relative risk for every study and for studies that reported the exposures in ranges we calculated the average of the upper and the lower cut-off point which was used as a midpoint. When the lowest or highest category was open-ended or had an extreme range we used the width of the adjacent interval to calculate an upper or lower confidence interval. A potential nonlinear dose–response relationship between BMI and waist circumference and risk of atrial fibrillation was examined by using fractional polynomial models [48]. We determined the best fitting second order fractional polynomial regression model, defined as the one with the lowest deviance. A likelihood ratio test was used to assess the difference between the nonlinear and linear models to test for nonlinearity [48].

Subgroup analyses stratified by sex, measurement versus self-report of adiposity measures, duration of follow-up, geographic location, number of cases, study quality scores, and adjustment for confounders (age, smoking, alcohol, and physical activity) and potential intermediates (hypertension, blood pressure, cholesterol, diabetes mellitus, coronary heart disease, heart failure, and left ventricular hypertrophy) were conducted to investigate potential sources of heterogeneity and heterogeneity between studies was quantitatively assessed by the Q test and I^2^ [49]. Meta-regression analyses were used to examine between subgroup differences in the summary estimates. Small study effects, such as publication bias, were assessed by inspecting the funnel plots for asymmetry and with Egger’s test [50] and Begg’s test [51] with the results considered to indicate small study effects when *p* < 0.10. Sensitivity analyses excluding one study at a time were conducted to clarify whether the results were simply due to one large study or a study with an extreme result.

## Results

**Fig. 1:**
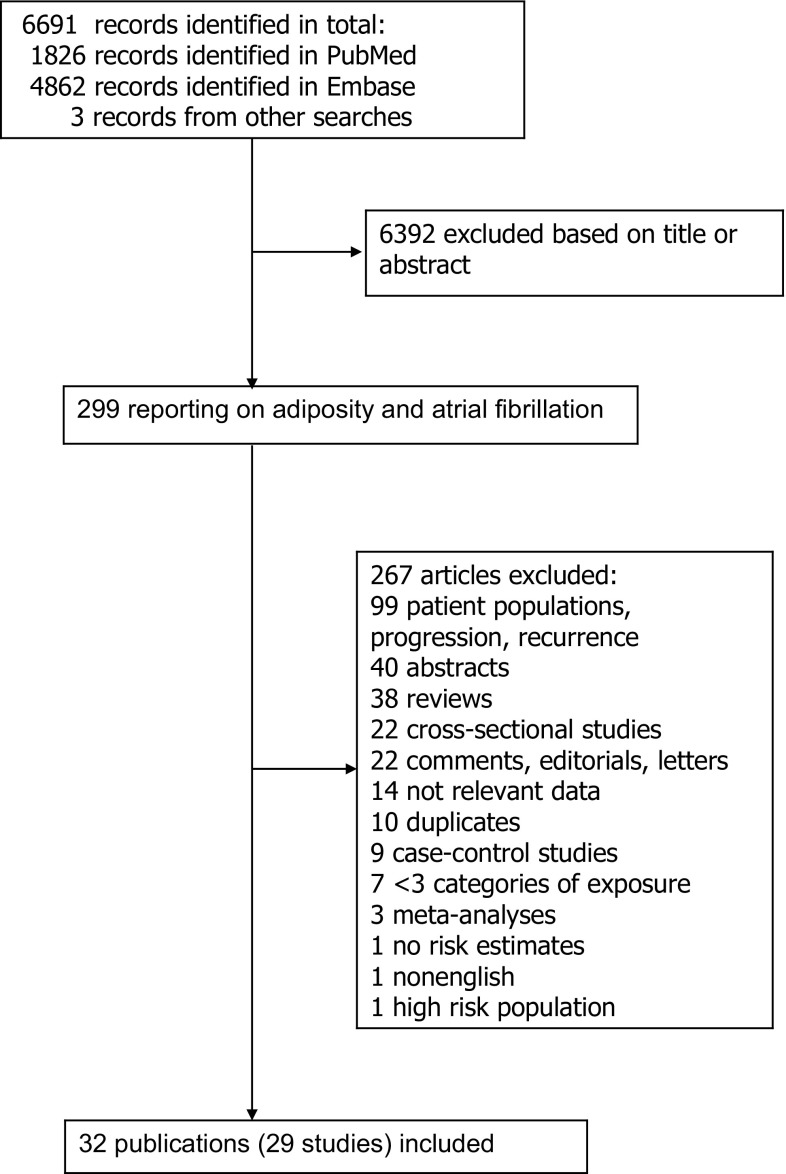
Flow-chart of study selection

We identified 29 prospective studies (32 publications) that were included in the systematic review of BMI, waist circumference, hip circumference, waist-to-hip ratio, body fat mass, body fat percentage, weight, and weight gain and risk of atrial fibrillation (Supplementary Table 4, Fig. 1) [18–34, 36–42, 52–59]. Only one study reported on pericardial fat, intrathoracic fat, and abdominal visceral fat and atrial fibrillation, thus it was not possible to conduct meta-analyses for these measures [52]. In addition, only one study reported on BMI and mortality from atrial fibrillation, thus the study was excluded from the main analysis, but it was included in a sensitivity analysis [54]. Fourteen studies were from Europe, eight were from the USA, four were from Asia, and three were from Australia (Supplementary Table 4).

### Body mass index

**Fig. 2:**
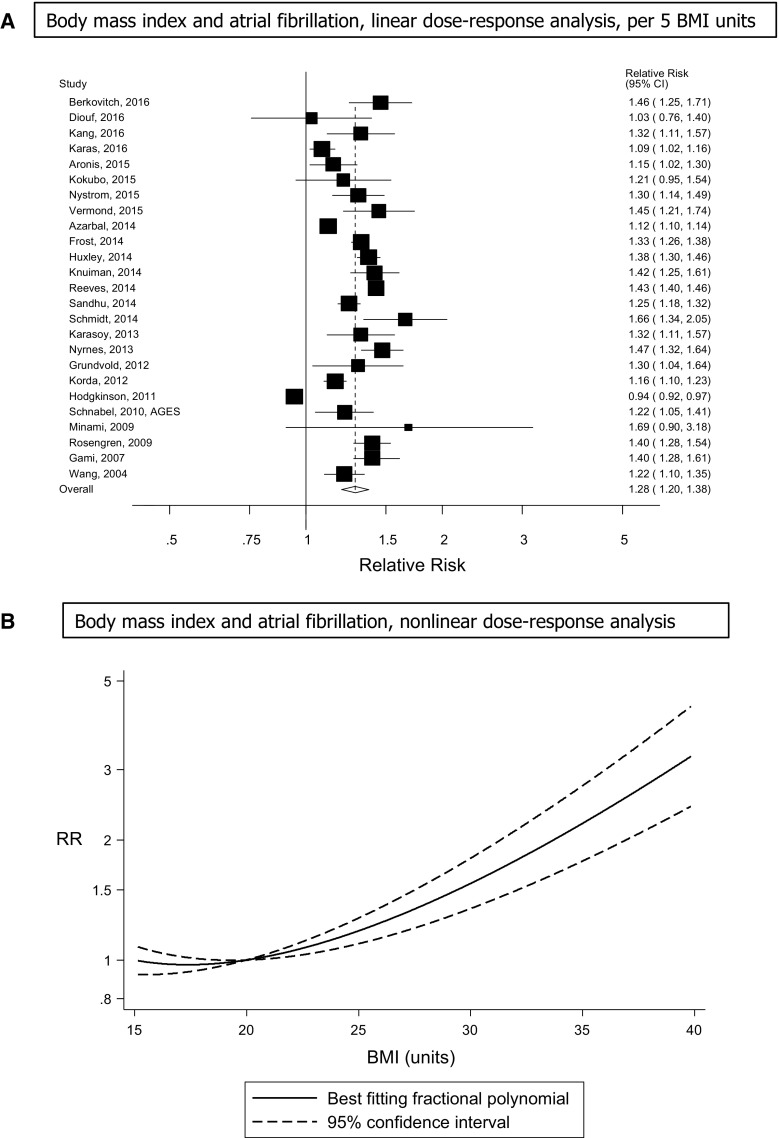
BMI and atrial fibrillation, linear and nonlinear dose–response analysis

Twenty-five prospective studies (25 publications) [18–21, 23–34, 36–40, 42, 55–57] including two nested case–control studies (were included in the dose–response analysis of BMI and atrial fibrillation incidence and included 83,006 incident cases among 2,405,381 participants. The summary RR for a 5 unit increment in BMI was 1.28 (95% confidence interval: 1.20–1.38, I^2^ = 97%, *p*_heterogeneity_ < 0.0001; Fig. 2a, Supplementary Table 7 ), and it was similar when stratified by gender, but the heterogeneity was lower among men (I^2^ = 37%) compared to women (I^2^ = 98%; Table 1). All but one of the studies found increased risk, but the strength of the association differed between studies. In sensitivity analyses excluding the most influential studies, the summary RR ranged from 1.27 (95% CI: 1.18–1.37) when excluding the Danish Military Conscripts study [28] to 1.30 (95% CI: 1.23–1.38) when excluding the UK General Practice Research Database Study [20]. There was no indication of publication bias with Egger’s test, *p* = 0.31, or with Begg’s test, *p* = 0.44, however, by inspection of the funnel plot there was some evidence of asymmetry with potentially smaller negative studies missing (Supplementary Fig. 1). There was evidence of a nonlinear association between BMI and atrial fibrillation, *p*_nonlinearity_ < 0.0001 (Fig. 2b, Supplementary Table 5) with a steeper increase in risk at higher BMI values. In a sensitivity analysis, one study of BMI and atrial fibrillation mortality [54] was included in the analysis, but the results remained similar, summary RR = 1.29 (95% CI: 1.20–1.38, I^2^ = 97%, *p*_heterogeneity_ < 0.0001) per 5 BMI units.

**Table 1:** Subgroup analyses of BMI and atrial fibrillation

		BMI				
		*n*	RR (95% CI)	*I* ^a^ (%)	*P*_h_^b^	*P*_h_^c^
All studies		25	1.28 (1.20–1.38)	96.8	<0.0001	
*Sex*						
Men		9	1.39 (1.30–1.48)	37.2	0.12	0.05/0.21
Women		7	1.30 (1.14–1.48)	98.1	<0.0001	
Men and women		11	1.25 (1.11–1.39)	94.3	<0.0001	
*Assessment of weight/height*						
Measured		19	1.27 (1.18–1.37)	95.2	<0.0001	0.66
Self-reported		4	1.28 (1.13–1.45)	94.9	<0.0001	
Not available		2	1.41 (1.26–1.58)	0	0.57	
*Duration of follow-up*						
<5 years		3	1.28 (1.12–1.47)	78.2	0.01	0.70
5 ≤ 10 years		7	1.37 (1.27–1.47)	38.8	0.13	
10 ≤ 15 years		8	1.23 (1.13–1.33)	91.0	<0.0001	
15 ≤ 20 years		3	1.33 (1.23–1.44)	72.3	0.03	
≥20 years		4	1.29 (0.96–1.73)	96.8	<0.0001	
*Geographic location*						
Europe		10	1.34 (1.16–1.56)	98.5	<0.0001	0.47
America		8	1.22 (1.14–1.31)	89.8	<0.0001	
Australia		3	1.22 (1.04–1.44)	78.1	0.01	
Asia		4	1.37 (1.23–1.52)	0	0.53	
*Number of cases*						
Cases < 250		5	1.38 (1.22–1.57)	43.4	0.13	0.10
Cases 250 ≤ 1000		10	1.32 (1.24–1.40)	47.2	0.05	
Cases ≥ 1000		10	1.23 (1.11–1.36)	98.7	<0.0001	
*Study quality*						
0–3		0				0.92
4–6		3	1.29 (1.08–1.54)	78.3	0.01	
7–9		22	1. 28 (1.19–1.38)	97.2	<0.0001	
*Adjustment for confounders*						
Age	Yes	23	1.27 (1.18–1.37)	97.0	<0.0001	0.24
	No	2	1.45 (1.15–1.83)	71.8	0.06	
Smoking	Yes	14	1.21 (1.11–1.32)	98.1	<0.0001	0.01
	No	11	1.40 (1.34–1.47)	0	0.56	
Alcohol	Yes	12	1.26 (1.11–1.42)	98.2	<0.0001	0.53
	No	13	1.30 (1.21–1.40)	86.1	<0.0001	
Physical activity	Yes	10	1.30 (1.18–1.43)	97.2	<0.0001	0.66
	No	15	1.28 (1.16–1.42)	94.3	<0.0001	
*Adjustment for potential intermediates*						
Hypertension	Yes	11	1.24 (1.13–1.35)	96.3	<0.0001	0.18
	No	14	1.33 (1.23–1.43)	88.0	<0.0001	
Blood pressure	Yes	7	1.26 (1.17–1.35)	32.4	0.18	0.76
	No	18	1.29 (1.19–1.40)	97.7	<0.0001	
Cholesterol	Yes	6	1.28 (1.15–1.43)	93.6	<0.0001	0.99
	No	19	1.29 (1.17–1.42)	97.2	<0.0001	
Diabetes mellitus	Yes	11	1.24 (1.14–1.36)	96.6	<0.0001	0.29
	No	14	1.32 (1.22–1.42)	88.2	<0.0001	
Coronary heart disease	Yes	13	1.27 (1.16–1.38)	96.3	<0.0001	0.58
	No	12	1.30 (1.20–1.41)	90.6	<0.0001	
Heart failure	Yes	8	1.18 (1.06–1.31)	96.7	<0.0001	0.02
	No	17	1.33 (1.26–1.41)	86.8	<0.0001	
Left ventricular hypertrophy	Yes	2	1.19 (1.10–1.28)	0	0.48	0.39
	No	23	1.29 (1.20–1.39)	97.1	<0.0001	

*n* denotes the number of studies

^a^ *P* for heterogeneity within each subgroup

^b^ *P* for heterogeneity between subgroups

^c^ *P* for heterogeneity between men and women (excluding men/women combined)

### Waist circumference

**Fig. 3:**
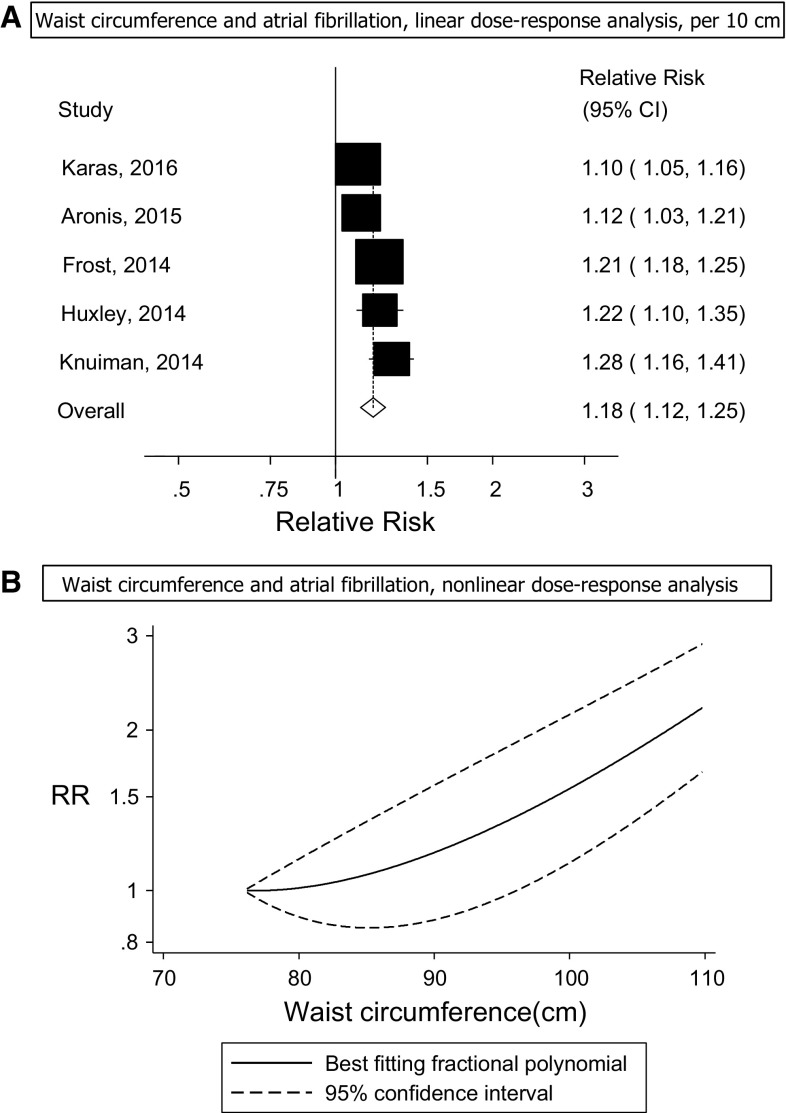
Waist circumference and atrial fibrillation, linear and nonlinear dose–response analysis

Five prospective studies (5 publications) [26, 29–32] were included in the analysis of waist circumference and risk of atrial fibrillation incidence and included 6120 cases among 80,752 participants. Three studies were from the USA, one from Denmark and one from Australia (Supplementary Table 4). The summary RR for a 10 cm increase in waist circumference was 1.18 (95% CI: 1.12–1.25, I^2^ = 73%, *p*_heterogeneity_ = 0.005) (Fig. 3a, Supplementary Table 7). The summary RR ranged from 1.16 (95% CI: 1.10–1.23) when the Busselton Health Study [29] was excluded to 1.20 (95% CI: 1.15–1.26) when the Cardiovascular Health Study [32] was excluded. There was no evidence of publication bias with Egger’s test, *p* = 0.85 or Begg’s test, *p* = 0.99, although the number of studies was limited. There was no evidence of a nonlinear association between waist circumference and atrial fibrillation incidence (*p*_nonlinearity_ = 0.09; Fig. 3b, Supplementary Table 6).

Waist-to-hip ratio, hip circumference, weight, body fat mass, body fat percentage, pericardial fat, intrathoracic fat, and abdominal visceral fat.

**Fig. 4:**
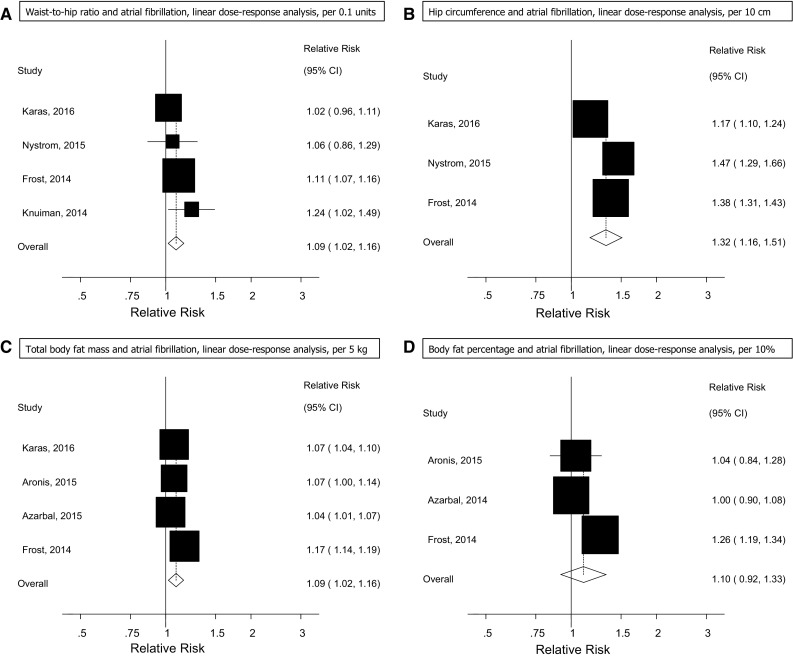
Waist-to-hip ratio, hip circumference, total body fat mass, and body fat percentage and atrial fibrillation

Four prospective studies (4 publications) [29, 30, 32, 33] were included in the analysis of waist-to-hip ratio and risk of atrial fibrillation (4259 cases and 67,837 participants) and the summary RR for a 0.1 unit increment in waist-to-hip ratio was 1.09 (95% CI: 1.02–1.16, I^2^ = 44%, p_heterogeneity_ = 0.15) (Fig. 4a, Supplementary Table 7).

Three prospective studies (3 publications) [30, 32, 33] were included in the analysis of hip circumference and risk of atrial fibrillation (3916 cases and 63,570 participants) and the summary RR for a 10 cm increase in hip circumference was 1.32 (95% CI: 1.16–1.51, I^2^ = 91%, *p*_heterogeneity_ < 0.0001; Fig. 4b, Supplementary Table 7).

Four prospective studies (3 publications) [29, 30, 32, 33] were included in the analysis of total body fat mass and atrial fibrillation (5037 cases and 71,098 participants), and the summary RR for a 5 kg increase in body fat mass was 1.09 (95% CI: 1.02–1.16, I^2^ = 94%, *p*_heterogeneity_ < 0.0001) (Fig. 4c, Supplementary Table 7).

Three prospective studies (3 publications) [29, 30, 33] were included in the analysis of body fat percentage and risk of atrial fibrillation (2952 cases and 57,990 participants) and the summary RR per 10% increase in fat percentage was 1.10 (95% CI: 0.92–1.33, I^2^ = 90% *p*_heterogeneity_ < 0.0001) (Fig. 4d, Supplementary Table 7).

**Fig. 5:**
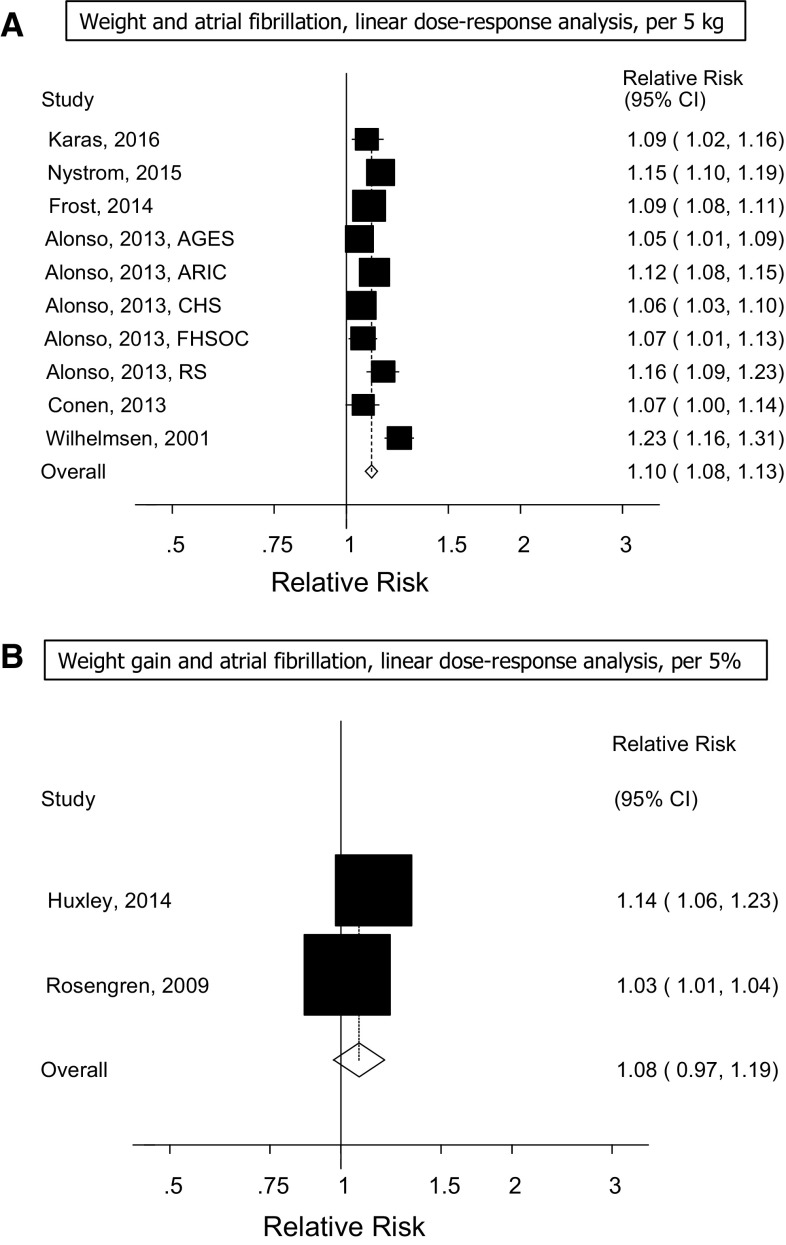
Weight and weight change and atrial fibrillation

Ten prospective studies (6 publications) [30, 32, 33, 53, 58, 59] were included in the analysis of weight and the risk of atrial fibrillation (7237 cases and 132,006 participants) and the summary RR for a 5 kg increment in weight was 1.10 (95% CI: 1.08–1.13, I^2^ = 74%, *p*_heterogeneity_ < 0.0001) (Fig. 5a, Supplementary Table 7). There was no evidence of publication bias with Egger’s test, *p* = 0.52, or Begg’s test, *p* = 0.59.

Two prospective studies [23, 26] were included in the analysis of weight gain and the risk of atrial fibrillation (3028 cases and 21,122 participants) and the summary RR was 1.08 (95% CI: 0.97–1.19, I^2^ = 86% *p*_heterogeneity_ = 0.007) (Fig. 5b) per 5% increase in weight gain. Only one study reported on pericardial fat, intrathoracic fat, abdominal visceral fat and the risk of atrial fibrillation and found hazard ratios of 1.13 (95% CI: 0.99–1.30), 1.19 (95% CI: 1.01–1.40), 1.09 (95% CI: 0.93–1.28), respectively [52].

### Subgroup and sensitivity analyses and study quality

The positive association between BMI, and risk of atrial fibrillation persisted in almost all subgroup analyses defined by gender, assessment of weight and height, duration of follow-up, geographic location, number of cases, study quality and adjustment for confounding and potential intermediate factors and there was little evidence of heterogeneity between any of these subgroups with meta-regression analyses (Table 1). In further subgroup analyses of two studies that reported data stratified by ethnicity [31, 37], the summary RR per 5 BMI units was 1.14 (95% CI: 1.06–1.22) among Caucasians and 1.23 (95% CI: 1.09–1.39) for African Americans, with no significant heterogeneity between subgroups, *p* = 0.39. When the studies of BMI and atrial fibrillation were stratified by study design, the summary RR was 1.15 (95% CI: 0.67–1.98, I^2^ = 69.5%) for the nested case–control studies and 1.30 (95% CI: 1.23–1.38, I^2^ = 94.3%) for the cohort studies. Study quality was high with a mean (median) score of 7.7 (8) out of 9 points in the analysis of BMI and atrial fibrillation.

## Discussion

This is to our knowledge the first meta-analysis to assess multiple adiposity measures in relation to risk of atrial fibrillation. There was a 28% increase in the relative risk per 5 units increase in BMI, a 18% increase in relative risk per 10 cm increase in waist circumference, a 9% increase in the relative risk per 0.1 unit increase in waist-to-hip ratio, a 32% increase in relative risk per 10 cm increase in hip circumference, a 9% increase in the relative risk per 5 kg increase in body fat mass, and a 10% increase in the relative risk per 5 kg increase in body weight, but no significant association was observed for body fat percentage or weight gain, although the number of studies was very low in these analyses. There was evidence of a nonlinear association between BMI and atrial fibrillation, with a slightly steeper association at higher BMI levels, however, there was evidence of increased risk even within the normal BMI range (22–24) compared to a BMI of around 20, although the increased risk was most pronounced in the obese and severely obese BMI ranges. The association between waist circumference and atrial fibrillation was approximately linear. The positive association between BMI and atrial fibrillation was observed across all geographic locations and both in Caucasians and African Americans, suggesting that adiposity is a risk factor for atrial fibrillation across populations.

The current findings are consistent with a previous meta-analysis of 5 cohort studies which found a 39 and 87% increase in the relative risk of atrial fibrillation among overweight and obese, respectively, compared to normal weight subjects [35]. However, the current analysis has a much larger number of studies and cases and participants (25 studies with 83,006 incident cases among 2,405,381 participants compared to 5 studies with 2114 cases and 78,602 participants) and thus provides a much more robust estimate of the association, in addition to a more comprehensive assessment of different adiposity measures in relation to risk of atrial fibrillation. Although a recent randomized trial did not find a statistically significant reduction in risk of atrial fibrillation among individuals with type 2 diabetes with a weight loss intervention, the study may have had too low power to detect a moderate reduction in risk [60]. The hazard ratio for the highest quintile of weight loss was 0.70 (95% CI: 0.41–1.18), thus a moderate reduction in risk cannot be excluded based on this trial. Another recent study of obese patients undergoing bariatric surgery found a reduced risk of developing atrial fibrillation with a hazard ratio of 0.71 (95% CI: 0.60–0.83) compared to the control group, providing additional evidence that adiposity is related to increased risk of atrial fibrillation [61]. Our findings of an increased risk of atrial fibrillation with higher hip circumference is somewhat in contrast to previous studies that have found an inverse association between hip circumference and cardiovascular disease [62], however, the inverse associations were only observed after further adjustment for BMI and waist circumference while none of the studies in the current meta-analysis made further adjustments for BMI and waist circumference. Further studies are therefore needed to clarify whether hip circumference, waist circumference and BMI are independently associated with atrial fibrillation.

Our meta-analysis has some limitations that need to be mentioned. Confounding by other risk factors may have influenced the results. However, the association between BMI and atrial fibrillation persisted in subgroup analyses when studies were stratified by whether they adjusted for confounding factors such as age, smoking, alcohol, and physical activity. In addition, the association persisted among studies that adjusted for potential intermediates including hypertension, blood pressure, serum cholesterol, diabetes, coronary heart disease, heart failure, and left ventricular hypertrophy. There was some evidence of heterogeneity between the subgroups of studies that adjusted for heart failure, with weaker, but still significant associations among studies with such adjustment. This could indicate that part of the association between adiposity and atrial fibrillation may be mediated by heart failure. This is consistent with our previous finding of an increased risk of heart failure related to both general and abdominal adiposity [7] and with the increased risk of atrial fibrillation among patients with heart failure [53]. Although the heterogeneity between studies was high, this appeared to be largely due to different effect sizes between studies, rather than differences in the direction of the association, as all but one study found a positive association. Exclusion of the study which showed an inverse association in the linear dose–response analysis did not substantially reduce the heterogeneity.

Measurements of weight, height, waist and hip circumferences may have been affected by measurement errors, however, the association for BMI was similar among studies that used measured weight and height compared to those that used self-reported weight and height. Validation studies have reported high correlations between self-reported and measured anthropometric measures [63–66]. BMI is an imperfect measure of body fatness as it does not distinguish between body fat and muscle mass. However, studies have shown high correlations between BMI and waist measures and body fat as measured by dual-energy X-ray absorptiometry (DXA) [67, 68]. Importantly, the association between adiposity and atrial fibrillation was in the direction of increased risk for all adiposity measures analysed, and the association with body fat mass (measured by DXA) did not appear to be stronger than that for BMI or waist circumference, supporting the use of these measures for the measurement of adiposity and for prediction of atrial fibrillation. Although publication bias or small study bias can affect the findings of meta-analyses of published literature, we found no evidence of such bias with Egger’s or Begg’s test. However, power was low for these tests in the analyses apart from BMI and weight because the number of studies was low.

Several potential mechanisms could explain an association between body fatness and risk of atrial fibrillation. Adiposity is associated with increased risk of hypertension [2], insulin resistance [69], diabetes [70], obstructive sleep apnea [71], coronary heart disease [72], and heart failure [7], which are established risk factors for atrial fibrillation [53, 73, 74]. Adiposity is associated with increased risk of left ventricular hypertrophy [75–77] and left atrial size [78, 79], and the latter may be due to hypertension, volume overload, left ventricular diastolic abnormalities, autonomic dysfunction and enhanced neurohormonal activation [80, 81]. In an experimental animal study weight gain resulted in atrial remodeling and increased atrial volumes, left atrial and systemic pressures, ventricular mass, pericardial fat volumes, increased atrial interstitial fibrosis, inflammation, myocardial lipid accumulation, and conduction abnormalities with slowing of atrial conduction and increased conduction heterogeneity [82]. Adiposity is related to low-grade inflammation [83, 84] which is strongly associated with atrial fibrillation [85]. Overweight and obesity is also related to greater epicardial fat thickness [86–88] which has been associated with alterations in atrial electrophysiology [89] and risk of atrial fibrillation [90, 91]. The findings of a recent Mendelian Randomisation study of genetic obesity and atrial fibrillation is consistent with a causal interpretation of the positive association found in the current meta-analysis between adiposity and atrial fibrillation [92].

Our meta-analysis has several strengths including the prospective design of the included studies which avoids recall bias and reduces the possibility for selection bias, the large number of cohort studies with >83,000 cases and >2.4 million participants in the BMI analysis which provided statistical power to detect moderate associations, the detailed dose–response analyses which clarified the shape of the dose–response relationship, the observation of a similar association between BMI and atrial fibrillation in different geographic regions, and robustness of the findings in multiple subgroup analyses as well as the high study quality of the included studies.

The findings have important clinical implications for the prevention of atrial fibrillation as a previous meta-analysis only analysed BMI, but not other fat measures in relation to the risk of atrial fibrillation [35], and have not assessed the dose–response relationship between adiposity and atrial fibrillation in as much detail as the current analysis. In addition, we found in subgroup analyses that higher BMI was associated with increased risk of atrial fibrillation in studies from Europe, North America, Australia and Asia, as well as in Caucasian and African American participants suggesting that avoidance of excess weight is important across populations. The current analysis suggests that both general and abdominal adiposity measures as well as increased hip circumference and total body fat mass is related to increased risk of atrial fibrillation and that being relatively slim as assessed by BMI, waist circumference and other adiposity measures may confer the lowest risk of atrial fibrillation. However, to what degree different fat measures independently of each other predict atrial fibrillation risk is not clear from the current data as few studies reported mutually adjusted results, but this requires further study. Because of the moderate number of studies in the analyses of other adiposity measures than BMI and weight further studies are needed of these measures. These findings have important public health implications because of the increasing prevalence of overweight and obesity worldwide [1] and because of the consistency of the results across populations. Thus if current trends continue unabated it might contribute to an increased incidence of atrial fibrillation and associated complications globally [16].

In conclusion, our findings confirm that overweight, obesity, abdominal fatness and high body fat mass increase the risk of atrial fibrillation.

## Electronic supplementary material

Below is the link to the electronic supplementary material.

Supplementary material 1 (PDF 414 kb)
