# Supplementary material for: Body mass index, abdominal fatness, fat mass and the risk of atrial fibrillation: a systematic review and dose–response meta-analysis of prospective studies
Source: Eur J Epidemiol. 2017 Feb 13;32(3):181–92. doi: 10.1007/s10654-017-0232-4 (PMC5380695; doi:10.1007/s10654-017-0232-4)
Supplement: Supplementary file 1 — Supplementary material 1 (PDF 414 kb) [file 10654_2017_232_MOESM1_ESM.pdf]

SUPPLEMENTAL MATERIAL

Aune D, Sen A, Schlesinger S, Norat T, Janszky I, Romundstad P, Tonstad S, Riboli E, Vatten LJ. Body mass index, abdominal fatness, fat mass and the risk of atrial fibrillation: a systematic review and dose-response meta-analysis of prospective studies: Eur J Epidemiol 2017; DOI: 10.1007/s10654-017-0232-4

Supplementary Table 1. PubMed search

1	"body mass index"
2	BMI
3	overweight
4	obesity
5	anthropometry
6	fatness
7	"body fatness"
8	"abdominal fatness"
9	"abdominal obesity"
10	"waist circumference"
11	"waist-to-hip ratio"
12	adiposity
13	"weight gain"
14	"weight change"
15	"weight loss"
16	"atrial fibrillation"
17	"atrial flutter"
18	1 OR 2 OR 3 OR 4 OR 5 OR 6 OR 7 OR 8 OR 9 OR 10 OR 11 OR 12 OR 13 OR 14 OR 15
19	16 OR 17
20	18 AND 19

Supplementary Table 2.Embase search

1	body mass index/
2	BMI/
3	overweight/
4	obesity/
5	anthropometry/
6	fatness/
7	body fatness/
8	abdominal fatness/
9	abdominal obesity/
10	waist circumference/
11	waist-to-hip ratio/
12	adiposity/
13	weight gain/
14	weight change/
15	weight loss/
16	atrial fibrillation/
17	atrial flutter/
18	case-control
19	cohort
20	cohorts
21	prospective
22	longitudinal
23	retrospective

24	follow-up
25	cross-sectional
26	hazard ratio
27	hazard ratios
28	relative risk
29	relative risks
30	incidence rate ratio
31	incidence rate ratios
32	odds ratio
33	odds ratios
34	incidence
35	1 OR 2 OR 3 OR 4 OR 5 OR 6 OR 7 OR 8 OR 9 OR 10 OR 11 OR 12 OR 13 OR 14 OR 15
36	16 OR 17
37	18 OR 19 OR 20 OR 21 OR 22 OR 23 OR 24 OR 25 OR 26 OR 27 OR 28 OR 29 OR 30 OR 31 OR 32 OR 33 OR 34
38	35 AND 36 AND 37

Supplementary Table 3.List of excluded studies and reasons for exclusion.

Exclusion reason	Reference number
Abstract	(1-40)
Case-control study	(41-49)
Cross-sectional study	(50-71)
Duplicates	(72-81)
High risk patients	(82)
Letter, News, comments, editorial	(83-104)
Meta-analysis	(105-107)
No risk estimates	(108)
Non-English	(109)
Not relevant data	(110-123)
Patient population, progression, recurrence	(124-222)
Review	(223-260)
<3 categories of exposure	(261-267)

Reference List

1. Djousse L. Association of modifiable lifestyle factors and risk of total and cardiovascular mortality among older us male physicians. Cardiology (Switzerland) Conference: International Academy of Cardiology 1920;July.

2. Alonso A, Lopez FL, Mac Lehose RF. Additive and multiplicative interactions of race with risk factors for atrial fibrillation: The Aric study. American Journal of Epidemiology Conference: 3rd North American Congress of Epidemiology Montreal, QC Canada Conference Start: 2011;01.

3. Grundvold I, Skretteberg PT, Kjeldsen SE, Arnesen H, Bodegard J. Physical fitness modifies the predictive impact of weight gain and body mass index for incident atrial fibrillation. Circulation Conference: American Heart Association's Scientific Sessions 2011;22.

4. Conen D, Glynn RJ, Sandhu RK, Tedrow UB, Albert CM. Differing risk factors for incident atrial fibrillation with and without left atrial enlargement. Circulation Conference: American Heart Association's Scientific Sessions 2011;22.

5. Kokubo Y, Shimizu W, Kamakura S et al. Obesity index and the risk of atrial fibrillation in an Urban Japanese cohort: The Suita study. Circulation Conference: American Heart Association's Scientific Sessions 2011;22.

6. Gruberg L, Parikh B, Malhotra A, Kort S. Impact of body mass index and gender on left atrial size and the presence of atrial fibrillation. Circulation Conference: American Heart Association's Scientific Sessions 2011;22.

7. Peng F, Lin J, Liu H. Metabolic syndrome and risk of atrial fibrillation in Chinese essential hypertensive patients. International Journal of Cardiology Conference: World Hypertension League Regional Congress 2011;October.

8. Sandhu RK, Conen D, Tedrow UB, Fitzgerald K, Glynn R, Albert CM. Risk factors associated with development of paroxysmal versus persistent atrial fibrillation. Circulation Conference: American Heart Association 2012;20.

9. Jensen PN, Thacker EL, Dublin S, Psaty BM, Heckbert SR. Racial differences in the incidence of and risk factors for atrial fibrillation in older adults: The cardiovascular health study. Circulation Conference: Epidemiology and Prevention/Physical Activity, Nutrition and Metabolism 2012;13.

10. Karasoy D, Gislason G, Torp-Pedersen C et al. Obesity is a powerful predictor of atrial fibrillation in fertile women. European Heart Journal Conference: ESC Congress 2012;August.

11. Kokubo Y, Shimizu W, Watanabe M et al. Impact of blood pressure and obesity on the risk of incident atrial fibrillation in the Suita Study: An urban cohort study. European Heart Journal Conference: ESC Congress 2012;August.

12. Sandhu R, Conen D, Tedrow UB, Glynn RJ, Albert CM. Risk factors associated with paroxysmal versus persistent/permanent atrial fibrillation. European Heart Journal Conference: ESC Congress 2012;August.

13. Karas MG, Yee L, Biggs ML et al. Central and general adiposity and risk of incident atrial fibrillation in older adults: The cardiovascular health study. Journal of the American College of Cardiology Conference: 61th Annual Scientific Session of the American College of Cardiology and i2 Summit: Innovation in Intervention, ACC 12 Chicago, IL United States Conference Start: 2012;27.

14. Willis BL, De FL, Radford N, Gao A, Leonard D, Berry J. Association of cardiorespiratory fitness and body mass index in midlife with atrial fibrillation in later life: The cooper center longitudinal study. Circulation Conference: American Heart Association 2013;26.

15. Schmidt M, Botker HE, Pedersen L, Sorensen HT. Obesity in young men and long-term risk of atrial fibrillation: 33-year follow-up of 12,850 young healthy men. Circulation Conference: American Heart Association 2013;26.

16. Neeland IJ, Turer A, Ayers C et al. Body fat distribution and incident cardiovascular disease in obese adults. Circulation Conference: American Heart Association 2013;26.

17. Pignatelli P, Pastori D, Cangemi R et al. Metabolic syndrome is an independent predictor of cardiovascular events in patients with atrial fibrillation. Circulation Conference: American Heart Association 2013;26.

18. Onuchina E, Solovyev O, Mochalova O, Onuchin S, Onuchina J. Risk factors of atrial fibrillation in patients with metabolic syndrome. Journal of Diabetes Conference: 5th International Congress on Prediabetes and the Metabolic Syndrome Early Interventions for Diabetes and Dysglycaemia Surgery in the Treatment of Obesity and Diabetes Vienna Austria Conference Start: 2013;April.

19. Grundvold I, Skretteberg PT, Liestoel K et al. Predictors of atrial fibrillation differ in men with high vs low physical fitness. European Heart Journal Conference: European Society of Cardiology, ESC Congress 2013;August.

20. Mahabadi AA, Lehmann N, Kalsch H et al. Epicardial adipose tissue and left atrial size as CT-derived predictors of atrial fibrillation: The heinz nixdorf recall study. European Heart Journal Conference: European Society of Cardiology, ESC Congress 2013;August.

21. Jamaly S, Jacobson P, Peltonen M, Carlsson L, Sjostrom L, Karason K. Obesity surgery and incidence of atrial fibrillation. European Heart Journal Conference: European Society of Cardiology, ESC Congress 2013;August.

22. Kelsey AM. Atrial fibrillation in postmenopausal women: Risk factors, diagnosis, and management. Menopause Conference: 25th Annual Meeting of the North American Menopause Society, NAMS 2014;December.

23. Kokubo Y, Kobayashi T, Watanbe M et al. A combination of metabolic syndrome components increased the risk of incident atrial fibrillation in a general urban cohort: The Suita study. Hypertension Conference: American Heart Association's High Blood Pressure Research 2014;November.

24. Vermond RA, Geelhoed B, Verweij N et al. Incidence of atrial fibrillation and relation with cardiovascular events, heart failure and mortality in a european community-based study-data of prevend. Circulation Conference: American Heart Association's 2014;25.

25. Berkovitch A, Kivity S, Klempfner R et al. Weight reduction is associated with reduced risk for atrial fibrillation in apparently healthy middle-age men and women. European Heart Journal Conference: European Society of Cardiology, ESC Congress 2014;01.

26. Grundvold I, Bodegard J, Nilsson PM et al. Body mass index and changes in weight are associated with risk of atrial fibrillation and cardiovascular mortality: A longitudinal cohort study of 7,169 patients with newly diagnosed type 2 diabetes.

European Heart Journal Conference: European Society of Cardiology, ESC Congress 2014;01.

27. Kokubo Y, Kobayashi T, Watanabe M et al. Combination of type 2 diabetes and overweight is linked to an increased incidence of atrial fibrillation in a general urban cohort population. European Heart Journal Conference: European Society of Cardiology, ESC Congress 2014;01.

28. Bulbulia R, Sherliker P, Clack R, Halliday A, Peto R, Lewington S. Adiposity, systolic blood pressure, smoking and atrial fibrillation: Analyses of 2.3 million us adults attending cardiovascular screening. Circulation Conference: American Heart Association's 2015;10.

29. Kokubo Y, Watanabe M, Higashiyama A et al. A risk score for the prediction of atrial fibrillation in the Japanese community: The Suita study. Circulation Conference: American Heart Association's 2015;10.

30. Nilsson PM, Zoller B, Sundquist J, Sundquist K. Predictors of severe obesity and associated morbidity in the Malmo Preventive Project: A population-based cohort study. Diabetologia Conference: 51st Annual Meeting of the European Association for the Study of Diabetes, EASD 2015;September.

31. Andersen K, Rasmussen F, Neovius M, Tynelius P, Sundstrom J. Anthropometric measures and risk of atrial fibrillation-a cohort study of 1.2 million young men. European Heart Journal Conference: European Society of Cardiology, ESC Congress 2015;01.

32. Nakamura S, Adachi H, Enomoto M et al. The trend of atrial fibrillation in the elderly men for 30 years in a japanese general population. Journal of Hypertension Conference: 25th European Meeting on Hypertension and Cardiovascular Protection, ESH 2015;June.

33. Aronis KN, Wang N, Phillips C et al. Associations of adiposity and atrial fibrillation in older adults: The health abc study. Circulation Conference: American Heart Association's Epidemiology and Prevention/Lifestyle and Cardiometabolic Health 2015;10.

34. Shulman EH, Kargoli F, Hoch E et al. Body mass index and the development of atrial fibrillation in hispanics, blacks and non-hispanic whites. Journal of the American College of Cardiology Conference: 65th Annual Scientific Session of the American College of Cardiology and i2 Summit: Innovation in Intervention, ACC 16 Chicago, IL United States Conference Start: 2016;05.

35. Baek Y, Yang P-S, Kim T-H et al. Associations of abdominal obesity and new onset atrial fibrillation in general population: A nationwide cohort study in Korea. Journal of the American College of Cardiology Conference: 65th Annual Scientific Session of the American College of Cardiology and i2 Summit: Innovation in Intervention, ACC 16 Chicago, IL United States Conference Start: 2016;05.

36. Heckbert SR, Wiggins KL, Blackshear C et al. Pericardial fat volume is associated with incident atrial fibrillation: The multi-ethnic study of atherosclerosis and the

jackson heart study. Circulation Conference: American Heart Association's Epidemiology and Prevention/Lifestyle and Cardiometabolic Health 2016;01.

37. Anderson JL, Knight S, May HT, Bunch TJ, Lappe DL, Muhlestein JB. Prevalence of atrial fibrillation (AF) risk factors and lone AF in a general healthcare population. Circulation Conference: American Heart Association 2012;20.

38. Wong CX, Sun MT, Mahajan R et al. Obesity and the risk of atrial fibrillation: A systematic review and meta-analysis. Circulation Conference: American Heart Association 2012;20.

39. Murakami C, Nagai T, Akira F et al. Total epicardial fat volume is associated with early recurrence of atrial fibrillation after catheter ablation. Journal of the American College of Cardiology Conference: 61th Annual Scientific Session of the American College of Cardiology and i2 Summit: Innovation in Intervention, ACC 12 Chicago, IL United States Conference Start: 2012;27.

40. White T, Cameron J, Mottram P. Weight confirmed as a risk factor for atrial fibrillation in 39,023 subjects. Heart Lung and Circulation Conference: Cardiac Society of Australia and New Zealand Annual Scientific Meeting and the International Society for Heart Research Australasian Section Annual Scientific Meeting 2015;2015.

41. Wong CX, Abed HS, Molaee P et al. Pericardial fat is associated with atrial fibrillation severity and ablation outcome. J Am Coll Cardiol 2011;57:1745-51.

42. Dublin S, French B, Glazer NL et al. Risk of new-onset atrial fibrillation in relation to body mass index. Arch Intern Med 2006;166:2322-8.

43. Al Chekakie MO, Welles CC, Metoyer R et al. Pericardial fat is independently associated with human atrial fibrillation. J Am Coll Cardiol 2010;56:784-8.

44. Batal O, Schoenhagen P, Shao M et al. Left atrial epicardial adiposity and atrial fibrillation. Circ Arrhythm Electrophysiol 2010;3:230-6.

45. Tsao H-M, Hu W-C, Wu M-H, Chen S-A. Abundance and distribution of epicardial adipose tissue surrounding the left atrium in patients with atrial fibrillation. Heart Rhythm Conference: 31st Annual Scientific Sessions of the Heart Rhythm Society, Heart Rhythm 2010;May.

46. Calvo N, Ramos P, Montserrat S et al. Emerging risk factors and the dose-response relationship between physical activity and lone atrial fibrillation: a prospective case-control study. Europace 2015.

47. Abed HS, Wong CX, Brooks AG et al. Periatrial fat volume is predictive of atrial fibrillation severity. Heart Rhythm Conference: 31st Annual Scientific Sessions of the Heart Rhythm Society, Heart Rhythm 2010;May.

48. Al Chekakie MON, Welles CC, Metoyer R et al. Pericardial fat is independently associated with human atrial fibrillation. Heart Rhythm Conference: 31st Annual Scientific Sessions of the Heart Rhythm Society, Heart Rhythm 2010;May.

49. Abed H, Wong C, Brooks A et al. Periatrial fat volume is predictive of atrial fibrillation severity. Heart Lung and Circulation Conference: New Zealand Annual Scientific Meeting of the Cardiac Society of Australia and New Zealand Adelaide, SA Australia Conference Start: 2010;2010.

50. Umetani K, Kodama Y, Nakamura T et al. High prevalence of paroxysmal atrial fibrillation and/or atrial flutter in metabolic syndrome. Circ J 2007;71:252-5.

51. Schnabel RB, Wilde S, Wild PS, Munzel T, Blankenberg S. Atrial fibrillation: its prevalence and risk factor profile in the German general population. Dtsch Arztebl Int 2012;109:293-9.

52. Lipworth L, Okafor H, Mumma MT et al. Race-specific impact of atrial fibrillation risk factors in blacks and whites in the southern community cohort study. American Journal of Cardiology 110 (11) (pp 1637-1642), 2012;01.

53. Schnabel RB, Johannsen SS, Wild PS, Blankenberg S. [Prevalence and risk factors of atrial fibrillation in Germany : data from the Gutenberg Health Study]. Herz 2015;40:8-15.

54. Martin-Rodriguez E, Guillen-Grima F, Marti A, Brugos-Larumbe A. Comorbidity associated with obesity in a large population: The APNA study. Obesity Research and Clinical Practice 9 (5) (pp 435-447), 2015;September.

55. Sun G, Guo L, Wang X et al. Prevalence of atrial fibrillation and its risk factors in rural China: A cross-sectional study. Journal of the American College of Cardiology Conference: 26th Great Wall International Congress of Cardiology, Asia Pacific Heart Congress 2015;20.

56. Guize L, Thomas F, Bean K, Benetos A, Pannier B. [Atrial fibrillation: prevalence, risk factors and mortality in a large French population with 15 years of follow-up]. Bull Acad Natl Med 2007;191:791-803.

57. Yap KB, Ng TP, Ong HY. Low prevalence of atrial fibrillation in community-dwelling Chinese aged 55 years or older in Singapore: a population-based study. Journal of Electrocardiology 41 (2) (pp 94-98), 2008;March.

58. Zhang X, Zhang S, Li Y et al. Association of obesity and atrial fibrillation among middle-aged and elderly Chinese. Int J Obes (Lond) 2009;33:1318-25.

59. Bonhorst D, Mendes M, Adragao P et al. Prevalence of atrial fibrillation in the Portuguese population aged 40 and over: the FAMA study. Rev Port Cardiol 2010;29:331-50.

60. Thanassoulis G, Massaro JM, O'Donnell CJ et al. Pericardial fat is associated with prevalent atrial fibrillation: the Framingham Heart Study. Circ Arrhythm Electrophysiol 2010;3:345-50.

61. Gill PS, Calvert M, Davis R, Davies MK, Freemantle N, Lip GY. Prevalence of heart failure and atrial fibrillation in minority ethnic subjects: the Ethnic-Echocardiographic Heart of England Screening Study (E-ECHOES). PLoS One 2011;6:e26710.

62. Long MJ, Jiang CQ, Lam TH et al. Atrial fibrillation and obesity among older Chinese: the Guangzhou Biobank Cohort Study. Int J Cardiol 2011;148:48-52.

63. Wetmore JB, Mahnken JD, Rigler SK et al. The prevalence of and factors associated with chronic atrial fibrillation in Medicare/Medicaid-eligible dialysis patients. Kidney Int 2012;81:469-76.

64. Schnabel RB, Wilde S, Wild PS, Munzel T, Blankenberg S. Atrial fibrillation: Its prevalence and risk factor profile in the German general population. Deutsches Arzteblatt International 109 (16) (pp 293-299), 2012;20.

65. Li Y, Wu YF, Chen KP et al. Prevalence of atrial fibrillation in China and its risk factors. Biomed Environ Sci 2013;26:709-16.

66. Hwan-Cheol P, Soon-Gil K, Kyoung KM, Bo YC, Jinho S. Prevalence of atrial fibrillation in asymptomatic healthy population in Korean rural area. Journal of Clinical Hypertension Conference: 28th Annual Scientific Meeting and Exposition of the American Society of Hypertension, Inc , ASH 2013;May.

67. Baena-Diez JM, Grau M, Fores R et al. Prevalence of atrial fibrillation and its associated factors in Spain: An analysis of 6 population-based studies. DARIOS Study. Rev Clin Esp 2014;214:505-12.

68. Chei CL, Raman P, Ching CK et al. Prevalence and Risk Factors of Atrial Fibrillation in Chinese Elderly: Results from the Chinese Longitudinal Healthy Longevity Survey. Chin Med J (Engl) 2015;128:2426-32.

69. Park HC, Park JK, Choi SI et al. Prevalence of Atrial Fibrillation and Relation to Echocardiographic Parameters in a Healthy Asymptomatic Rural Korean Population. J Korean Med Sci 2015;30:1078-84.

70. Sun GZ, Guo L, Wang XZ et al. Prevalence of atrial fibrillation and its risk factors in rural China: a cross-sectional study. Int J Cardiol 2015;182:13-7.

71. Svetlana Shalnova SA, Deev AD, Konstantinov VV. Prevalence of atrial fibrillation and its association with traditional risk factors and coronary heart disease in Russian population. European Journal of Preventive Cardiology Conference: EuroPRevent 2013;April.

72. Frost L, Hune LJ, Vestergaard P. [Overweight, obesity and risk factors for atrial fibrillation or flutter--secondary publication. The cohort study Diet, Cancer and Health]. Ugeskr Laeger 2005;167:3507-9.

73. Rodriguez F, Stefanick ML, Greenland P et al. Racial and ethnic differences in atrial fibrillation risk factors and predictors in women: Findings from the Women's Health Initiative. Am Heart J 2016;176:70-7.

74. Huxley RR, Lopez FL, Folsom AR et al. Absolute and attributable risks of atrial fibrillation in relation to optimal and borderline risk factors: the Atherosclerosis Risk in Communities (ARIC) study. Circulation 2011;123:1501-8.

75. Schnabel RB, Sullivan LM, Levy D et al. Development of a risk score for atrial fibrillation (Framingham Heart Study): a community-based cohort study. Lancet 2009;373:739-45.

76. Son MK, Lim NK, Cho MC, Park HY. Incidence and Risk Factors for Atrial Fibrillation in Korea: the National Health Insurance Service Database (2002-2010). Korean Circ J 2016;46:515-21.

77. Perez MV, Wang PJ, Larson JC et al. Risk factors for atrial fibrillation and their population burden in postmenopausal women: the Women's Health Initiative Observational Study. Heart 2013;99:1173-8.

78. Ermakov S, Azarbal F, Stefanick ML et al. The associations of leptin, adiponectin and resistin with incident atrial fibrillation in women. Heart 2016.

79. Tedrow UB, Conen D, Ridker PM et al. The long- and short-term impact of elevated body mass index on the risk of new atrial fibrillation the WHS (women's health study). J Am Coll Cardiol 2010;55:2319-27.

80. Ruigomez A, Johansson S, Wallander MA, Rodriguez LA. Incidence of chronic atrial fibrillation in general practice and its treatment pattern. J Clin Epidemiol 2002;55:358-63.

81. Toren K, Schioler L, Soderberg M, Giang KW, Rosengren A. The association between job strain and atrial fibrillation in Swedish men. Occup Environ Med 2015;72:177-80.

82. Casaclang-Verzosa G, Barnes ME, Blume G et al. C-reactive protein, left atrial volume, and atrial fibrillation: a prospective study in high-risk elderly. Echocardiography 2010;27:394-9.

83. Osorio J. Atrial fibrillation: Obesity increases risk of AF in women. Nat Rev Cardiol 2010;7:417.

84. Dagres N, nastasiou-Nana M. Atrial fibrillation and obesity an association of increasing importance. J Am Coll Cardiol 2010;55:2328-9.

85. Conen D. Obesity and atrial fibrillation: the evidence is gaining weight. Europace 2013;15:771-2.

86. Chahal CAA, Somers VK. Risk factors: Sleep apnoea, atrial fibrillation, and heart failure - Quo vadis? Nature Reviews Cardiology 12 (5) (pp 263-264), 2015;28.

87. Ergun G, Basaran O, Dogan V, Dogan MM, Biteker M. Obesity and atrial fibrillation. Int J Cardiol 2016;223:159-60.

88. Coromilas J. Obesity and atrial fibrillation: is one epidemic feeding the other? JAMA 2004;292:2519-20.

89. Liew R. Obstructive sleep apnea, obesity, and atrial fibrillation--what is the mechanistic link? Heart Rhythm 2012;9:1417-8.

90. Hatem SN. Atrial Fibrillation and Obesity: Not Just a Coincidence. J Am Coll Cardiol 2015;66:12-3.

91. Korantzopoulos P, Kolettis TM. Obesity and the risk of new-onset atrial fibrillation. JAMA 2005;293:1974.

92. Parish D. Obesity and the risk of new-onset atrial fibrillation. JAMA 2005;293:1974.

93. Bajaj HS, Hillson SD. Obesity and the risk of new-onset atrial fibrillation. JAMA 2005;293:1974-5.

94. Obesity is a risk factor for new-onset atrial fibrillation. Geriatrics and Aging 8 (2) (pp 9), 2005;February.

95. Mascitelli L, Pezzetta F. Obesity, inflammation, and risk of atrial fibrillation or flutter. Am J Med 2006;119:e9.

96. Arias MA, Sanchez-Gila J. Obesity as a risk factor for developing postoperative atrial fibrillation. Chest 2006;129:828-9.

97. Cheng TO. Obesity as a risk factor for atrial fibrillation. Int J Cardiol 2007;114:145.

98. Arias MA, Sanchez AM, onso-Fernandez A, Garcia-Rio F. Atrial fibrillation, obesity, and obstructive sleep apnea [3]. Archives of Internal Medicine 167 (14) (pp 1552-1553), 2007;23.

99. Badheka AO, Rathod A, Bharadwaj A, Afonso L, Jacob S. Obesity paradox in outcomes of atrial fibrillation. American Journal of Cardiology 108 (3) (pp 474), 2011;01.

100. Deshmukh A, Pant S, Kumar G, Badheka AO, Paydak H. Impact of obesity on atrial fibrillation hospitalization. Int J Cardiol 2012;159:241-2.

101. Dhein S. Fat and the Heart: A More and More Complex Interplay. Thoracic and Cardiovascular Surgeon 62 (7) (pp 543-546), 2014;25.

102. Liu F, Li Y, Xu Y. Weight loss to prevent atrial fibrillation: The role of epicardial adipose tissue. Int J Cardiol 2016;204:124-5.

103. Amar D. Obesity as a risk factor for developing postoperative atrial fibrillation [6]. Chest 129 (3) (pp 828-829), 2006;March.

104. Girerd N, Pibarot P, Mathieu P. Predictors of atrial fibrillation following coronary artery bypass surgery: Increased waist circumference rather than increased body mass index? Medical Science Monitor 17 (3) (pp 23), 2011;2011.

105. Wanahita N, Messerli FH, Bangalore S, Gami AS, Somers VK, Steinberg JS. Atrial fibrillation and obesity--results of a meta-analysis. Am Heart J 2008;155:310-5.

106. Wong CX, Sullivan T, Sun MT et al. Obesity and the risk of incident, post-operative, and post-ablation atrial fibrillation: A meta-analysis of 626,603 individuals in 51 studies. JACC: Clinical Electrophysiology 1 (3) (pp 139-152), 2015;June.

107. Wong C, Brooks A, Sun M et al. Obesity and the risk of atrial fibrillation: A systematic review and meta-analysis. Heart Lung and Circulation Conference: Cardiac Society of Australia and New Zealand Annual Scientific Meeting and the International Society for Heart Research Australasian Section Annual Scientific Meeting 2012;2012.

108. Uyarel H, Onat A, Yuksel H, Can G, Ordu S, Dursunoglu D. Incidence, prevalence, and mortality estimates for chronic atrial fibrillation in Turkish adults. [Turkish]. Turk Kardiyoloji Dernegi Arsivi 36 (4) (pp 214-222), 2008;June.

109. Shi JH, Ji CP, Xing AJ et al. [Impact of combined systolic blood pressure and body mass index on the risk of new-onset atrial fibrillation]. Zhonghua Xin Xue Guan Bing Za Zhi 2016;44:231-7.

110. Kang S. Research in brief. The Lancet Diabetes and Endocrinology 4 (7) (pp 565), 2016;01.

111. Sliwa K, Carrington MJ, Klug E et al. Predisposing factors and incidence of newly diagnosed atrial fibrillation in an urban African community: Insights from the Heart of Soweto Study. Heart 96 (23) (pp 1878-1882), 2010;December.

112. Djousse L, Wilk JB, Hanson NQ, Glynn RJ, Tsai MY, Gaziano JM. Association Between Adiponectin and Heart Failure Risk in the Physicians' Health Study. Obesity (no pagination), 2012;15.

113. Petrone AB, Gaziano JM, Djousse L. Chocolate consumption and risk of heart failure in the Physicians' Health Study. European Journal of Heart Failure 16 (12) (pp 1372-1376), 2014;01.

114. Perkiomaki J, Ukkola O, Kiviniemi A et al. Heart rate variability findings as a predictor of atrial fibrillation in middle-aged population. Journal of Cardiovascular Electrophysiology 25 (7) (pp 719-724), 2014;July.

115. Blomstrand A, Blomstrand C, Ariai N, Bengtsson C, Bjorkelund C. Stroke incidence and association with risk factors in women: A 32-year follow-up of the Prospective Population Study of Women in Gothenburg. BMJ Open 4 (10) (no pagination), 2014;e005173.

116. Huynh K. Atrial fibrillation. Weight loss reduces AF burden. Nat Rev Cardiol 2015;12:260.

117. Rodriguez CJ, Soliman EZ, Alonso A et al. Atrial fibrillation incidence and risk factors in relation to race-ethnicity and the population attributable fraction of atrial fibrillation risk factors: The Multi-Ethnic Study of Atherosclerosis. Annals of Epidemiology 25 (2) (pp 71-76), 2015;01.

118. Macheret F, Bartz TM, Djousse L et al. Higher circulating adiponectin levels are associated with increased risk of atrial fibrillation in older adults. Heart 101 (17) (pp 1368-1374), 2015;01.

119. Tiwari S, Schirmer H, Jacobsen BK et al. Association between diastolic dysfunction and future atrial fibrillation in the Tromso Study from 1994 to 2010. Heart 101 (16) (pp 1302-1308), 2015;01.

120. Qureshi WT, Alirhayim Z, Blaha MJ et al. Cardiorespiratory fitness and risk of incident atrial fibrillation results from the henry ford exercise testing (FIT) project. Circulation 131 (21) (pp 1827-1834), 2015;26.

121. Pandit SV, Anumonwo J, Jalife J. Atrial Fibrillation Susceptibility in Obesity: An Excess Adiposity and Fibrosis Complicity? Circ Res 2016;118:1468-71.

122. Schmidt M, Ulrichsen SP, Pedersen L, Botker HE, Nielsen JC, Sorensen HT. 30-year nationwide trends in incidence of atrial fibrillation in Denmark and associated 5-year risk of heart failure, stroke, and death. International Journal of Cardiology 225 (pp 30-36), 2016;15.

123. Cui F, Zhang L, Yu C, Hu S, Zhang Y. Estimation of the disease burden attributable to 11 risk factors in Hubei Province, China: A comparative risk assessment. International Journal of Environmental Research and Public Health 13 (10) (no pagination), 2016;944.

124. Wong YW, Thomas L, Sun J-L et al. Predictors of incident heart failure hospitalizations among patients with impaired glucose tolerance insight from the nateglinide and valsartan in impaired glucose tolerance outcomes research study. Circulation: Heart Failure 6 (2) (pp 203;March.

125. Zacharias A, Schwann TA, Riordan CJ, Durham SJ, Shah AS, Habib RH. Obesity and risk of new-onset atrial fibrillation after cardiac surgery. Circulation 2005;112:3247-55.

126. Arias MA, Sanchez AM, Sanchez-Gila J. Postoperative atrial fibrillation and obesity. Am J Cardiol 2006;97:1551-2.

127. Echahidi N, Mohty D, Pibarot P et al. Obesity and metabolic syndrome are independent risk factors for atrial fibrillation after coronary artery bypass graft surgery. Circulation 2007;116:I213-I219.

128. Novo G, Mansueto P, La Franca ML et al. Risk factors, atrial fibrillation and thromboembolic events. Int Angiol 2008;27:433-8.

129. Jongnarangsin K, Chugh A, Good E et al. Body mass index, obstructive sleep apnea, and outcomes of catheter ablation of atrial fibrillation. J Cardiovasc Electrophysiol 2008;19:668-72.

130. Mont L, Tamborero D, Elosua R et al. Physical activity, height, and left atrial size are independent risk factors for lone atrial fibrillation in middle-aged healthy individuals. Europace 2008;10:15-20.

131. Tang RB, Liu XH, Kalifa J et al. Body mass index and risk of left atrial thrombus in patients with atrial fibrillation. Am J Cardiol 2009;104:1699-703.

132. Nicolaou VN, Papadakis JE, Dermitzakis G, Dermitzaki SI, Tsakiris AK. Effect of obesity on atrial size in older women with non-valvular paroxysmal atrial fibrillation. Aging Clin Exp Res 2009;21:344-8.

133. Tang RB, Gao LY, Dong JZ et al. Metabolic syndrome in patients with atrial fibrillation in the absence of structural heart disease from a tertiary hospital in China. Chin Med J (Engl) 2009;122:2744-7.

134. Girerd N, Pibarot P, Fournier D et al. Middle-aged men with increased waist circumference and elevated C-reactive protein level are at higher risk for postoperative atrial fibrillation following coronary artery bypass grafting surgery. Eur Heart J 2009;30:1270-8.

135. Filardo G, Hamilton C, Hamman B, Hebeler RF, Jr., Grayburn PA. Relation of obesity to atrial fibrillation after isolated coronary artery bypass grafting. Am J Cardiol 2009;103:663-6.

136. Soliman EZ, Prineas RJ, Go AS et al. Chronic kidney disease and prevalent atrial fibrillation: the Chronic Renal Insufficiency Cohort (CRIC). Am Heart J 2010;159:1102-7.

137. Uchiyama S, Shibata Y, Hirabayashi T et al. Risk factor profiles of stroke, myocardial infarction, and atrial fibrillation: a Japanese Multicenter Cooperative Registry. J Stroke Cerebrovasc Dis 2010;19:190-7.

138. Chilukuri K, Dalal D, Gadrey S et al. A prospective study evaluating the role of obesity and obstructive sleep apnea for outcomes after catheter ablation of atrial fibrillation. J Cardiovasc Electrophysiol 2010;21:521-5.

139. Suzuki S, Yamashita T, Ohtsuka T et al. Body size and atrial fibrillation in Japanese outpatients. Circ J 2010;74:66-70.

140. Ahmed I, Almquist AK, Henry TD et al. Obesity and pulmonary hypertension are independent risk factors for postoperative paroxysmal atrial fibrillation after heart valve replacement surgery. Heart Rhythm Conference: 31st Annual Scientific Sessions of the Heart Rhythm Society, Heart Rhythm 2010;May.

141. Onuchina EL, Solov'ev OV, Mochalova OV, Kononov SK, Onuchin SG. [Metabolic syndrome and chronic persistent atrial fibrillation]. Klin Med (Mosk) 2011;89:26-31.

142. Bramer S, van Straten AH, Soliman Hamad MA, Berreklouw E, van den Broek KC, Maessen JG. Body mass index predicts new-onset atrial fibrillation after cardiac surgery. Eur J Cardiothorac Surg 2011;40:1185-90.

143. Guglin M, Maradia K, Chen R, Curtis AB. Relation of obesity to recurrence rate and burden of atrial fibrillation. Am J Cardiol 2011;107:579-82.

144. Thacker EL, McKnight B, Psaty BM et al. Association of body mass index, diabetes, hypertension, and blood pressure levels with risk of permanent atrial fibrillation. J Gen Intern Med 2013;28:247-53.

145. Cai L, Yin Y, Ling Z et al. Predictors of late recurrence of atrial fibrillation after catheter ablation. Int J Cardiol 2013;164:82-7.

146. Letsas KP, Siklody CH, Korantzopoulos P et al. The impact of body mass index on the efficacy and safety of catheter ablation of atrial fibrillation. Int J Cardiol 2013;164:94-8.

147. Zethelius B, Gudbjornsdottir S, Eliasson B, Eeg-Olofsson K, Svensson A-M, Cederholm J. Risk factors for atrial fibrillation in type 2 diabetes. Report from the Swedish National Diabetes Register: NDR. Diabetologia Conference: 50th Annual Meeting of the European Association for the Study of Diabetes, EASD 2014;September.

148. Abed HS, Nelson AJ, Richardson JD et al. Impact of weight reduction on pericardial adipose tissue and cardiac structure in patients with atrial fibrillation. American Heart Journal 169 (5) (pp 655-662 e2), 2015;01.

149. Guenancia C, Stamboul K, Garnier F et al. Obesity and new-onset atrial fibrillation in acute myocardial infarction: A gender specific risk factor. Archives of Cardiovascular Diseases Supplements Conference: 25es Journees Europeennes de la Societe Francaise de Cardiologie Paris France Conference Start: 2015;January.

150. Abed HS, Nelson AJ, Richardson JD et al. Impact of weight reduction on pericardial fat volume and cardiac structure: Implications for atrial fibrillation in a randomized clinical trial. European Heart Journal Conference: European Society of Cardiology, ESC Congress 2015;01.

151. Abed HS, Nelson AJ, Richardson JD, Worthley SG, Wittert A, Leong DP. Impact of weight reduction on pericardial fat volume and cardiac structure: Implications for atrial fibrillation-a randomized clinical trial. Heart Rhythm Conference: 36th Annual Scientific Sessions of the Heart Rhythm Society, Heart Rhythm 2015;May.

152. Lavie CJ, De SA, Parto P et al. Obesity and Prevalence of Cardiovascular Diseases and Prognosis-The Obesity Paradox Updated. Prog Cardiovasc Dis 2016;58:537-47.

153. Banach M, Goch A, Misztal M, Rysz J, Jaszewski R, Goch JH. Predictors of paroxysmal atrial fibrillation in patients undergoing aortic valve replacement. Journal of Thoracic and Cardiovascular Surgery 134 (6) (pp 1569-1576), 2007;December.

154. Tsang TSM, Barnes ME, Miyasaka Y et al. Obesity as a risk factor for the progression of paroxysmal to permanent atrial fibrillation: A longitudinal cohort study of 21 years. European Heart Journal 29 (18) (pp 2227-2233), 2008;September.

155. Badheka A, Rathod A, Kizilbash M et al. An obesity paradox in outcomes of atrial fibrillation? Heart Rhythm Conference: 30th Annual Scientific Sessions of the Heart Rhythm Society, Heart Rhythm 2009;May.

156. Badheka AO, Rathod A, Kizilbash MA et al. Influence of Obesity on Outcomes in Atrial Fibrillation: Yet Another Obesity Paradox. American Journal of Medicine 123 (7) (pp 646-651), 2010;July.

157. Garadah T, Gabani S, Alawi MA, bu-Taleb A. Prevalence and Predisposing Factors of Atrial Fibrillation in a Multi-Ethnic Society: The Impact of Racial Differences in Bahrain. Open J Cardiovasc Surg 2011;4:9-16.

158. Needleman M, Calkins H. Atrial fibrillation ablation in obese patients. Cardiac Electrophysiology Clinics 4 (3) (pp 327-334), 2012;September.

159. Sotomi Y, Inoue K, Ito N et al. Incidence and risk factors for very late recurrence of atrial fibrillation after radiofrequency catheter ablation. Europace 2013;15:1581-6.

160. Overvad TF, Rasmussen LH, Skjoth F, Overvad K, Lip GY, Larsen TB. Body mass index and adverse events in patients with incident atrial fibrillation. Am J Med 2013;126:640-17.

161. Guijian L, Jinchuan Y, Rongzeng D, Jun Q, Jun W, Wenqing Z. Impact of body mass index on atrial fibrillation recurrence: a meta-analysis of observational studies. Pacing Clin Electrophysiol 2013;36:748-56.

162. Shoemaker MB, Muhammad R, Farrell M et al. Relation of morbid obesity and female gender to risk of procedural complications in patients undergoing atrial fibrillation ablation. Am J Cardiol 2013;111:368-73.

163. Wang J, Shao X-H, Zhang H et al. The influence of body mass index on outcomes in patients with atrial fibrillation: The obesity paradox. Circulation Conference: American Heart Association 2013;26.

164. Zhang H, Yang Y, Zhu J et al. Body mass index and mortality in patients with atrial fibrillation. Cardiology (Switzerland) Conference: China Heart Congress International Heart Forum 2013;September.

165. Fioravanti F, Brisinda D, Sorbo AR, Fenici R. BMI Reduction Decreases AF Recurrence Rate in a Mediterranean Cohort. J Am Coll Cardiol 2015;66:2264-5.

166. Hamatani Y, Ogawa H, Uozumi R et al. Low Body Weight Is Associated With the Incidence of Stroke in Atrial Fibrillation Patients - Insight From the Fushimi AF Registry. Circ J 2015;79:1009-17.

167. Inoue H, Kodani E, Atarashi H, Okumura K, Yamashita T, Origasa H. Impact of Body Mass Index on the Prognosis of Japanese Patients With Non-Valvular Atrial Fibrillation. Am J Cardiol 2016.

168. Bunch TJ, May HT, Bair TL et al. Long-term influence of body mass index on cardiovascular events after atrial fibrillation ablation. J Interv Card Electrophysiol 2016.

169. Proietti M, Lane DA, Lip GY. Relation of Nonvalvular Atrial Fibrillation to Body Mass Index (from the SPORTIF Trials). Am J Cardiol 2016.

170. Phan K, Khuong JN, Xu J, Kanagaratnam A, Yan TD. Obesity and postoperative atrial fibrillation in patients undergoing cardiac surgery: Systematic review and meta-analysis. Int J Cardiol 2016;217:49-57.

171. Sandhu RK, Ezekowitz J, Andersson U et al. The 'obesity paradox' in atrial fibrillation: observations from the ARISTOTLE (Apixaban for Reduction in Stroke and Other Thromboembolic Events in Atrial Fibrillation) trial. Eur Heart J 2016.

172. Chu CY, Lee WH, Hsu PC et al. Association of Increased Epicardial Adipose Tissue Thickness With Adverse Cardiovascular Outcomes in Patients With Atrial Fibrillation. Medicine (Baltimore) 2016;95:e2874.

173. Kwon Y, Norby FL, Jensen PN et al. Association of Smoking, Alcohol, and Obesity with Cardiovascular Death and Ischemic Stroke in Atrial Fibrillation: The Atherosclerosis Risk in Communities (ARIC) Study and Cardiovascular Health Study (CHS). PLoS One 2016;11:e0147065.

174. Senoo K, Lip GY. Body Mass Index and Adverse Outcomes in Elderly Patients With Atrial Fibrillation: The AMADEUS Trial. Stroke 2016;47:523-6.

175. Wang J, Yang YM, Zhu J et al. Overweight is associated with improved survival and outcomes in patients with atrial fibrillation. Clin Res Cardiol 2014;103:533-42.

176. Wong C, Brooks A, Sun M et al. Body mass index predicts postoperative atrial fibrillation: Results of a contemporary meta-analysis. Heart Lung and Circulation Conference: Cardiac Society of Australia and New Zealand Annual Scientific Meeting and the International Society for Heart Research Australasian Section Annual Scientific Meeting 2012;2012.

177. Wright GA, Thomas K, Gula L. Is body habitus a risk factor for atrial fibrillation and does it affect outcome of AF ablation? Canadian Journal of Cardiology Conference: 66th Annual Meeting of the Canadian Cardiovascular Society Montreal, QC Canada Conference Start: 2013;October.

178. Adrian VH, Kaw R, Pasupuleti V et al. Association between obesity and postoperative at rial fibrillation in patients undergoing cardiac surgery: A systematic review and meta-analysis. Journal of the American College of Cardiology Conference: 62nd Annual Scientific Session of the American College of Cardiology and i2 Summit: Innovation in Intervention, ACC 13 San Francisco, CA United States Conference Start: 2013;12.

179. Black MH, Wu J, Singer DE et al. Body mass index and outcomes among patients with atrial fibrillation from the atria2-CVRN study. Circulation: Cardiovascular Quality and Outcomes Conference: American Heart Association's Quality of Care and Outcomes Research in Cardiovascular Disease and Stroke 2013;May.

180. Wong CX, Sun MT, Pathak R et al. Body mass index predicts postoperative atrial fibrillation: Results of a contemporary meta-analysis. Circulation Conference: American Heart Association 2012;20.

181. Zhuang J, Lu Y, Tang K, Peng W, Xu Y. Influence of body mass index on recurrence and quality of life in atrial fibrillation patients after catheter ablation: a meta-analysis and systematic review. Clin Cardiol 2013;36:269-75.

182. Wang J, Yang YM, Zhu J, Zhang H, Shao XH. Obesity paradox in patients with atrial fibrillation and heart failure. Int J Cardiol 2014;176:1356-8.

183. Abed HS, Wittert GA, Leong DP et al. Effect of weight reduction and cardiometabolic risk factor management on symptom burden and severity in patients with atrial fibrillation: a randomized clinical trial. JAMA 2013;310:2050-60.

184. Jideus L, Blomstrom P, Nilsson L, Stridsberg M, Hansell P, Blomstrom-Lundqvist C. Tachyarrhythmias and triggering factors for atrial fibrillation after coronary artery bypass operations. Annals of Thoracic Surgery 69 (4) (pp 1064-1069), 2000;April.

185. Knyazer B, Abramov D, Bilenko N, Ganiel A, Ishay Y, Katz A. Incidence and predictors of atrial fibrillation after coronary artery bypass grafting in the Negev--are we different from the rest of the world?. [Hebrew]. Harefuah 147 (4) (pp 299-304, 375), 2008;Apr.

186. Golmohammadi M, Javid GE, Farajzadeh H. Incidence and risk factors for atrial fibrillation after first coronary artery bypass grafting in Urumiyeh imam Khomeini Hospital from 2006 to 2008. Iranian Cardiovascular Research Journal 4 (2) (pp 86-90), 2010;2010.

187. Sun X, Boyce SW, Hill PC et al. Association of body mass index with new-onset atrial fibrillation after coronary artery bypass graft surgery. Cardiovascular Revascularization Medicine Conference: Cardiovascular Research Technologies, CRT 2010;July-September.

188. Tadic M, Ivanovic B, Zivkovic N. Predictors of atrial fibrillation following coronary artery bypass surgery. Medical Science Monitor 17 (1) (pp CR48-CR55), 2011;2011.

189. Efird JT, Gudimella P, O'Neal WT et al. Comparison of Risk of Atrial Fibrillation in Black Versus White Patients After Coronary Artery Bypass Grafting. Am J Cardiol 2016;117:1095-100.

190. Sun X, Boyce SW, Hill PC et al. Association of body mass index with new-onset atrial fibrillation after coronary artery bypass grafting operations. Ann Thorac Surg 2011;91:1852-8.

191. Pan W, Hindler K, Lee V-V, Vaughn WK, Collard CD. Obesity in diabetic patients undergoing coronary artery bypass graft surgery is associated with increased postoperative morbidity. Anesthesiology 104 (3) (pp 441-447), 2006;March.

192. Ho KM, Bertenshaw C, Same S et al. Differential associations between body mass index and outcomes after elective adult cardiac surgery: a linked data cohort study. Anaesth Intensive Care 2013;41:573-83.

193. Hernandez AV, Kaw R, Pasupuleti V et al. Association between obesity and postoperative atrial fibrillation in patients undergoing cardiac operations: a systematic review and meta-analysis. Ann Thorac Surg 2013;96:1104-16.

194. Verdecchia P, Dagenais G, Healey J et al. Blood pressure and other determinants of new-onset atrial fibrillation in patients at high cardiovascular risk in the Ongoing Telmisartan Alone and in Combination with Ramipril Global Endpoint Trial/Telmisartan Randomized AssessmeNt Study in ACE iNtolerant subjects with cardiovascular Disease studies. Journal of Hypertension 30 (5) (pp 1004-1014), 2012;May.

195. Grundvold I, Bodegard J, Nilsson PM et al. Body weight and risk of atrial fibrillation in 7,169 patients with newly diagnosed type 2 diabetes; an observational study. Cardiovasc Diabetol 2015;14:5.

196. ves-Cabratosa L, Garcia-Gil M, Comas-Cufi M et al. Diabetes and new-onset atrial fibrillation in a hypertensive population. Ann Med 2016;48:119-27.

197. Zethelius B, Eliasson B, Eeg-Olofsson K, Svensson AM, Gudbjornsdottir S, Cederholm J. A new model for 5-year risk of cardiovascular disease in type 2 diabetes, from the Swedish National Diabetes Register (NDR). Diabetes Res Clin Pract 2011;93:276-84.

198. Arisoy E, Korkmaz I, Eren SH et al. Analyses of the risc factors for Atrial fibrillation among the patients who were admitted to emergency service. Journal of Clinical and Analytical Medicine 3 (2) (no pagination), 2012;April.

199. Liu HL, Lu XL, Guo ZP, Lin JX. [Association between metabolic syndrome and incidence of atrial fibrillation in essential hypertensive patients without left ventricular hypertrophy]. Zhonghua Xin Xue Guan Bing Za Zhi 2010;38:15-9.

200. Karajamaki AJ, Patsi OP, Savolainen M, Kesaniemi YA, Huikuri H, Ukkola O. Non-Alcoholic Fatty Liver Disease as a Predictor of Atrial Fibrillation in Middle-Aged Population (OPERA Study). PLoS One 2015;10:e0142937.

201. Acharya M, Harling L, Moscarelli M, Ashrafian H, Athanasiou T, Casula R. Influence of body mass index on outcomes after minimal-access aortic valve replacement through a J-shaped partial upper sternotomy. J Cardiothorac Surg 2016;11:74.

202. Guenancia C, Stamboul K, Garnier F et al. Obesity and new-onset atrial fibrillation in acute myocardial infarction: a gender specific risk factor. Int J Cardiol 2014;176:1039-41.

203. Gizurarson S, Stahlman M, Jeppsson A et al. Atrial fibrillation in patients admitted to coronary care units in western Sweden - focus on obesity and lipotoxicity. J Electrocardiol 2015;48:853-60.

204. Zethelius B, Gudbjornsdottir S, Eliasson B, Eeg-Olofsson K, Svensson AM, Cederholm J. Risk factors for atrial fibrillation in type 2 diabetes: report from the Swedish National Diabetes Register (NDR). Diabetologia 2015;58:2259-68.

205. Gao M, Sun J, Young N et al. Impact of Body Mass Index on Outcomes in Cardiac Surgery. Journal of Cardiothoracic and Vascular Anesthesia 30 (5) (pp 1308-1316), 2016;01.

206. Sumeray M, Steiner M, Sutton P, Treasure T. Age and obesity as risk factors in perioperative atrial fibrillation. Lancet 2 (8608) (pp 448), 1988;1988.

207. Zhu W, Wan R, Liu F et al. Relation of Body Mass Index With Adverse Outcomes Among Patients With Atrial Fibrillation: A Meta-Analysis and Systematic Review. J Am Heart Assoc 2016;5.

208. Ivanovic B, Tadic M, Bradic Z, Zivkovic N, Stanisavljevic D, Celic V. The influence of the metabolic syndrome on atrial fibrillation occurrence and outcome after coronary bypass surgery: A 3-year follow-up study. Thoracic and Cardiovascular Surgeon 62 (7 Supplement 01) (pp 561-568), 2014;01.

209. Garimella RS, Sears SF, Gehi AK. Depression and Physical Inactivity as Confounding the Effect of Obesity on Atrial Fibrillation. Am J Cardiol 2016;117:1760-4.

210. Haghjoo M, Salem N, Rafati M, Fazelifar A. Predictors of the atrial fibrillation following catheter ablation of typical atrial flutter. Res Cardiovasc Med 2013;2:90-4.

211. Im SI, Chun KJ, Park SJ, Park KM, Kim JS, On YK. Long-term Prognosis of Paroxysmal Atrial Fibrillation and Predictors for Progression to Persistnt or Chronic Atrial Fibrillation in the Korean Population. J Korean Med Sci 2015;30:895-902.

212. Pawlowska-Jenerowicz W, Dabrowski M. The metabolic syndrome as a risk factor for idiopathic paroxysmal atrial fibrillation: A one year follow-up after a first episode of atrial fibrillation. European Heart Journal Conference: European Society of Cardiology, ESC Congress 2009;September.

213. Menzorov M, Shutov A, Serov V, Menzorova.E. Anthropometric parameters and early reccurence of atrial fibrillation. European Journal of Internal Medicine Conference: 10th Congress of the European Federation of Internal Medicine Athens Greece Conference Start: 2011;October.

214. Nigam A, Khairy P, Talajic M, Levesque S, Roy D. The Influence of body-mass index on outcomes in subjects with atrial fibrillation and heart failure: Data from the AF-CHF study. European Heart Journal Conference: European Society of Cardiology, ESC Congress 2011;August.

215. Lim T, Koay C, See V et al. Larger body size but not BMI predict longer atrial fibrillation ablation procedure times and increased arrhythmia recurrences on long term follow up. Heart Lung and Circulation Conference: Cardiac Society of Australia and New Zealand Annual Scientific Meeting and the International Society for Heart Research Australasian Section Annual Scientific Meeting 2011;2011.

216. Kim T-H, Park J, Park J-K et al. Pericardial fat volume is an independent predictor for clinical recurrence of atrial fibrillation and obstructive sleep apnea in patients who underwent catheter ablation: Over 600 case study. Circulation Conference: American Heart Association 2013;26.

217. Babar T, Mekuria S, Malik P, Chung MK. Obesity and recurrence of atrial fibrillation after cardioversion. Journal of the American College of Cardiology Conference: American College of Cardiology 58th Annual Scientific Session and i2 Summit: Innovation in Intervention Orlando, FL United States Conference Start: 2009;2009.

218. Smit MD, Groenveld HF, Achekar ID, Wiesfeld AC, Van G, I. Body mass index predicts subacute and overall atrial fibrillation recurrence in patients with early persistent atrial fibrillation. Heart Rhythm Conference: 31st Annual Scientific Sessions of the Heart Rhythm Society, Heart Rhythm 2010;May.

219. Guglin M, Chen R, Maradia K. Obesity is associated with higher recurrence of atrial fibrillation. European Heart Journal Conference: European Society of Cardiology, ESC Congress 2010;September.

220. Ferrer-Hita JJ, Rodriguez-Gonzalez A, Blanco-Palacios G et al. Statins therapy, body max index and short and long-term prediction of atrial fibrillation recurrence after electrical cardioversion. Journal of Cardiovascular Electrophysiology Conference: Venice Arrhythmias 2011;October.

221. Vatutin M, Kalinkina N, Shevelok A, Kashanskaya O. Obesity as a risk factor for atrial fibrillation recurrence. Journal of Interventional Cardiac Electrophysiology Conference: 8th Annual Congress of the European Cardiac Arrhythmia Society, ECAS 2012;April.

222. Siebermair J, Clauss S, Schussler F et al. Role of hemodynamic parameters and body mass index in patients undergoing pulmonary vein isolation for treatment of atrial fibrillation. Journal of Interventional Cardiac Electrophysiology Conference: 9th Annual Congress of the European Cardiac Arrhythmia Society, ECAS 2013;April.

223. Lavie CJ, Milani RV, Ventura HO. Obesity and Cardiovascular Disease. Risk Factor, Paradox, and Impact of Weight Loss. Journal of the American College of Cardiology 53 (21) (pp 1925;26.

224. Gersh BJ, Tsang TS, Seward JB. The changing epidemiology and natural history of nonvalvular atrial fibrillation: clinical implications. Trans Am Clin Climatol Assoc 2004;115:149-59.

225. Gersh BJ, Tsang TSM, Barnes ME, Seward JB. The changing epidemiology of non-valvular atrial fibrillation: The role of novel risk factors. European Heart Journal, Supplement 7 (C) (pp C5-C11), 2005;May.

226. Wolk R, Somers VK. Obesity-related cardiovascular disease: Implications of obstructive sleep apnea. Diabetes, Obesity and Metabolism 8 (3) (pp 250-260), 2006;May.

227. Chen LY, Shen WK. Epidemiology of atrial fibrillation: a current perspective. Heart Rhythm 2007;4:S1-S6.

228. Somers VK, Gami AS, Block PC. Incident atrial fibrillation: Impact of obstructive sleep apnea and obesity. ACC Cardiosource Review Journal 16 (5) (pp 6-8), 2007;May.

229. Anand RG, Peters RW, Donahue TP. Obesity and dysrhythmias. Journal of the cardiometabolic syndrome 3 (3) (pp 149-154), 2008;2008.

230. Conen D, Osswald S, Albert CM. Epidemiology of atrial fibrillation. Swiss Med Wkly 2009;139:346-52.

231. Rosiak M, Dziuba M, Chudzik M et al. Risk factors for atrial fibrillation: Not always severe heart disease, not always so 'lonely'. Cardiol J 2010;17:437-42.

232. Kupczynska K, Wierzbowska-Drabik K. Obesity and cardiovascular risk - Current communications review. [Polish]. Polski Przeglad Kardiologiczny 12 (2) (pp 149-153), 2010;2010.

233. Kuhne M, Conen D. Atrial fibrillation in women. Therapy 7 (2) (pp 139-146), 2010;March.

234. Musil V. Risk factors of atrial fibrillation. [Slovak]. Kardiologicka Revue 12 (3) (pp 131-133), 2010;2010.

235. Mattioli AV. Lifestyle and atrial fibrillation. Expert Rev Cardiovasc Ther 2011;9:895-902.

236. Needleman M, Calkins H. The role of obesity and sleep apnea in atrial fibrillation. Curr Opin Cardiol 2011;26:40-5.

237. Artham SM, Lavie CJ, De SA, Ventura HO, Milani RV. Obesity, Age, and Cardiac Risk. Current Cardiovascular Risk Reports 5 (2) (pp 128-137), 2011;2011.

238. Lioni L, Korantzopoulos P, Letsas KP. Catheter ablation of atrial fibrillation in overweight and obese patients. Journal of Atrial Fibrillation 2 (8) (no pagination), 2011;December.

239. Zalesin KC, Franklin BA, Miller WM, Peterson ED, McCullough PA. Impact of Obesity on Cardiovascular Disease. Medical Clinics of North America 95 (5) (pp 919-937), 2011;September.

240. Asghar O, Alam U, Hayat SA, Aghamohammadzadeh R, Heagerty AM, Malik RA. Obesity, diabetes and atrial fibrillation; epidemiology, mechanisms and interventions. Curr Cardiol Rev 2012;8:253-64.

241. Schnabel RB. Can we predict the occurrence of atrial fibrillation? Clin Cardiol 2012;35 Suppl 1:5-9.

242. Al Chekakie MO, Akar JG. Epicardial fat and atrial fibrillation: A review. Journal of Atrial Fibrillation 2 (10) (no pagination), 2012;April.

243. Menezes AR, Lavie CJ, DiNicolantonio JJ et al. Cardiometabolic risk factors and atrial fibrillation. Rev Cardiovasc Med 2013;14:e73-e81.

244. Abed HS, Wittert GA. Obesity and atrial fibrillation. Obes Rev 2013;14:929-38.

245. Magnani JW, Hylek EM, Apovian CM. Obesity begets atrial fibrillation: a contemporary summary. Circulation 2013;128:401-5.

246. Menezes AR, Lavie CJ, DiNicolantonio JJ et al. Atrial fibrillation in the 21st century: a current understanding of risk factors and primary prevention strategies. Mayo Clin Proc 2013;88:394-409.

247. Menezes AR, Lavie CJ, DiNicolantonio JJ et al. Atrial fibrillation in the 21st century: A current understanding of risk factors and primary prevention strategies. Mayo Clinic Proceedings 88 (4) (pp 394-409), 2013;April.

248. Tadic M, Ivanovic B, Cuspidi C. What do we currently know about metabolic syndrome and atrial fibrillation? Clinical Cardiology 36 (11) (pp 654-662), 2013;November.

249. Asghar O, Alam U, Hayat SA, Aghamohammadzadeh R, Heagerty AM, Malik RA. Diabetes, obesity and atrial fibrillation: Epidemiology, mechanisms and interventions. Journal of Atrial Fibrillation 6 (2) (pp 47-55), 2013;August-September.

250. Rahman F, Kwan GF, Benjamin EJ. Global epidemiology of atrial fibrillation. Nature Reviews Cardiology 11 (11) (pp 639-654), 2014;25.

251. Andrade J, Khairy P, Dobrev D, Nattel S. The clinical profile and pathophysiology of atrial fibrillation: Relationships among clinical features, epidemiology, and mechanisms. Circulation Research 114 (9) (pp 1453-1468), 2014;25.

252. Nalliah CJ, Sanders P, Kottkamp H, Kalman JM. The role of obesity in atrial fibrillation. Eur Heart J 2015.

253. Menezes AR, Lavie CJ, De SA et al. Lifestyle Modification in the Prevention and Treatment of Atrial Fibrillation. Prog Cardiovasc Dis 2015;58:117-25.

254. Goudis CA, Korantzopoulos P, Ntalas IV, Kallergis EM, Ketikoglou DG. Obesity and atrial fibrillation: A comprehensive review of the pathophysiological mechanisms and links. J Cardiol 2015;66:361-9.

255. Miller JD, Aronis KN, Chrispin J et al. Obesity, Exercise, Obstructive Sleep Apnea, and Modifiable Atherosclerotic Cardiovascular Disease Risk Factors in Atrial Fibrillation. J Am Coll Cardiol 2015;66:2899-906.

256. Mahajan R, Lau DH, Sanders P. Impact of obesity on cardiac metabolism, fibrosis, and function. Trends in Cardiovascular Medicine 25 (2) (pp 119-126), 2015;01.

257. Wong CX, Ganesan AN, Selvanayagam JB. Epicardial fat and atrial fibrillation: current evidence, potential mechanisms, clinical implications, and future directions. Eur Heart J 2016.

258. Duraj I, Broncel M. [Obesity as a risk factor for atrial fibrillation]. Pol Merkur Lekarski 2016;40:5-8.

259. Hatem SN, Redheuil A, Gandjbakhch E. Cardiac adipose tissue and atrial fibrillation: the perils of adiposity. Cardiovasc Res 2016;109:502-9.

260. Iacobellis G. Is obesity a risk factor for atrial fibrillation? Nat Clin Pract Cardiovasc Med 2005;2:134-5.

261. Watanabe H, Tanabe N, Watanabe T et al. Metabolic syndrome and risk of development of atrial fibrillation: the Niigata preventive medicine study. Circulation 2008;117:1255-60.

262. Chamberlain AM, Agarwal SK, Ambrose M, Folsom AR, Soliman EZ, Alonso A. Metabolic syndrome and incidence of atrial fibrillation among blacks and whites in the Atherosclerosis Risk in Communities (ARIC) Study. Am Heart J 2010;159:850-6.

263. Smith JG, Platonov PG, Hedblad B, Engstrom G, Melander O. Atrial fibrillation in the Malmo Diet and Cancer study: a study of occurrence, risk factors and diagnostic validity. Eur J Epidemiol 2010;25:95-102.

264. Tanner RM, Baber U, Carson AP et al. Association of the metabolic syndrome with atrial fibrillation among United States adults (from the REasons for Geographic and Racial Differences in Stroke [REGARDS] Study). Am J Cardiol 2011;108:227-32.

265. Suzuki H, Ohira T, Takeishi Y et al. Increased prevalence of atrial fibrillation after the Great East Japan Earthquake: Results from the Fukushima Health Management Survey. Int J Cardiol 2015;198:102-5.

266. O'Neal WT, Judd SE, Limdi NA et al. Differential Impact of Risk Factors in Blacks and Whites in the Development of Atrial Fibrillation: the Reasons for Geographic And Racial Differences in Stroke (REGARDS) Study. J Racial Ethn Health Disparities 2016.

267. Alonso A, Bahnson JL, Gaussoin SA et al. Effect of an intensive lifestyle intervention on atrial fibrillation risk in individuals with type 2 diabetes: The Look AHEAD randomized trial. Am Heart J 2015;170:770-7.

Supplementary Table 4: Prospective studies of adiposity and atrial fibrillation risk

Author, publication year, country/ region	Study name	Recruitment and follow-up period	Study size, gender, age, number of cases	Assessment of weight and height	Exposure by subgroup	Description of quantiles of categories	RR (95% CI)	Adjustment for confounders
Wilhelmsen L et al, 2001, Sweden	The Multifactor Primary Prevention Study	1970-1973 – 1996, 25.2 years follow-up	7495 men, age 47-55 years: 754 AF cases	Measured	Body weight	≤72.9 kg<br>73.0-83.9<br>≥84.0	1.00<br>1.58 (1.28-1.93)<br>2.19 (1.78-2.69)	Age
Wang TJ et al, 2004, USA	Framingham Heart Study	1979-1982/1979-1983 – 1999, 13.7 years follow-up	5282 men and women, age 35-90 years: 526 AF cases	Measured	BMI, men<br><br><br><br>BMI, women<br><br><br><br>BMI, men<br><br><br><br>BMI, women	<25.0<br>25.0-<30.0<br>≥30.0<br>Per 1 unit<br><25.0<br>25.0-<30.0<br>≥30.0<br>Per 1 unit<br><25.0<br>25.0-<30.0<br>≥30.0<br>Per 1 unit<br><25.0<br>25.0-<30.0<br>≥30.0<br>Per 1 unit	1.00<br>1.09 (0.82-1.43)<br>1.49 (1.06-2.09)<br>1.04 (1.01-1.07)<br>1.00<br>1.20 (0.90-1.62)<br>1.59 (1.13-2.22)<br>1.04 (1.01-1.07)<br>1.00<br>1.10 (0.84-1.46)<br>1.52 (1.09-2.13)<br>1.04 (1.01-1.07)<br>1.00<br>1.13 (0.84-1.52)<br>1.46 (1.03-2.07)<br>1.04 (1.01-1.07)	Age, SBP, use of antihypertensive therapy, DM, ECG –LVH, prior MI or CHF, cigarette smoking, significant heart murmur<br><br><br><br><br><br><br><br>+adjusted for interim MI/CHF
Frost L et al, 2005, Denmark	Danish Diet, Cancer, and Health Study	1993-2001, 5.7 years follow-up	22482 men and 25107 women, age 50-64 years: 553 AF cases	Measured	BMI, men<br><br><br><br>BMI, women	18.5-25.0<br>25.0-30.0<br>≥30.0<br>Per 1 unit<br>18.5-25.0<br>25.0-30.0<br>≥30.0<br>Per 1 unit	1.00<br>1.75 (1.35-2.27)<br>2.35 (1.70-3.25)<br>1.08 (1.05-1.11)<br>1.00<br>1.39 (0.99-1.94)<br>1.99 (1.31-3.02)<br>1.06 (1.03-1.09)	Age, height, length of education, smoking status, alcohol, total cholesterol, hypertension treatment, SBP
Murphy NF et al, 2006, United Kingdom	Renfrew-Paisley Study	1972-1976 - NA, 20 years follow-up	15402 men and women, age 45-64 years: 5 AF deaths	Measured	BMI, all<br>BMI, men<br>BMI, women<br>BMI, all	Per unit<br>Per unit<br>Per unit<br>18.5-24.9	1.08 (0.94-1.25)<br>1.04 (0.52-2.06)<br>1.07 (0.92-1.25)<br>1.00	Age, sex (all), adjusted FEV1, cigarettes per day, social class

					 <br><br>BMI, men<br><br><br><br>BMI, women	25.0-29.9<br>≥30.0<br>Per unit<br>18.5-24.9<br>25.0-29.9<br>≥30.0<br>Per unit<br>18.5-24.9<br>25.0-29.9<br>≥30.0<br>Per unit	1.12 (0.80-1.57)<br>1.64 (1.07-2.51)<br>1.05 (1.01-1.08)<br>1.00<br>1.19 (0.74-1.92)<br>0.96 (0.42-2.20)<br>0.99 (0.93-1.06)<br>1.00<br>1.02 (0.63-1.64)<br>2.02 (1.20-3.39)<br>1.06 (1.02-1.11)	
Gami AS et al, 2007, USA	Olmsted County Study	1987-2003, 4.7 years follow-up	3542 men and women, mean age 49 years: 133 AF cases	Unclear	BMI	Per 1 unit	1.07 (1.05-1.10)	Age, sex, CAD, lowest nocturnal oxygen saturation
Minami M et al, 2009, Japan	Ishikawa Prefecture	1998-2006, NA	69 AF cases (men) 138 controls (nested case-control study)	Unclear	BMI	Per 1 unit	1.11 (0.98-1.26)	Age, time period, SBP, TCH, gamma-glutamyltranspeptidase, uric acid, fasting plasma glucose, hemoglobin, cardiomegaly, Brinkman index, alcohol
Rosengren A et al, 2009, Sweden	The Multifactor Primary Prevention Study	1970-1973 - 2004, 34.3 years follow-up	6903 men, age 47-56 years: 1253 AF hospital discharge diagnosis cases	Measured (current)<br>Self-reported (weight at age 20 years)	BMI at age 20<br><br><br><br><br>Midlife BMI<br><br><br><br><br><br><br>Body surface area<br><br><br><br>Weight change from age 20 to midlife	<21<br>21-22.2<br>22.3-23.7<br>>23.7<br>Per 1 unit<br><20.00<br>20.00-22.49<br>22.50-24.99<br>25.00-27.49<br>27.50-29.99<br>≥30.00<br>Per 1 unit<br><1.75 m^2<br>1.75-1.83<br>1.83-1.91<br>>1.91<br>Loss of more than 4%<br>-4 to +4%<br>+5-15%<br>+16-35%	1.00<br>1.02 (0.87-1.20)<br>1.06 (0.91-1.25)<br>1.28 (1.10-1.50)<br>1.05 (1.02-1.08)<br>1.11 (0.67-1.78)<br>1.00<br>1.15 (0.94-1.41)<br>1.32 (1.10-1.66)<br>1.50 (1.21-1.88)<br>1.56 (1.20-2.02)<br>1.04 (1.02-1.06)<br>1.00<br>1.39 (1.16-1.66)<br>1.52 (1.28-1.81)<br>1.95 (1.65-2.31)<br>1.11 (0.84-1.47)<br>1.00<br>1.08 (0.90-1.30)<br>1.22 (1.02-1.47)	Age<br><br><br><br><br>Age, intercurrent heart failure, intercurrent MI, SBP, treatment for hypertension, smoking, diabetes, alcohol, occupational class

						+>35%	1.31 (1.02-1.68)	
Schnabel RB et al, 2010, Iceland	Age, Gene/ Environment Susceptibility-Reykjavik Study	2002-2006 – 2008, 5 years follow-up	4238 men and women, age 45-95 years: 226 AF cases	Measured	BMI	Per 5 units	1.22 (1.05-1.41)	Age, sex, SBP, hypertension treatment, PR interval, prevalent heart failure
Schnabel RB et al, 2010, USA	Cardiovascular Health Study	1989-1990/ 1992-1993 – 2005, 5 years follow-up	5410 men and women, age 45-95 years: 958 AF cases	Measured	BMI, whites<br>BMI, African Americans	Per 5 units<br>Per 5 units	1.14 (1.05-1.23)<br>1.29 (1.10-1.51)	Age, sex, SBP, hypertension treatment, PR interval, valvular heart disease (whites only), prevalent heart failure
Hodgkinson JA et al, 2011, United Kingdom	UK General Practice Research Database	1987-2007, NA	44348 AF cases 161104 controls (nested case-control study)	Measured	BMI	10-19<br>20-24<br>25-29<br>≥30	1.14 (1.09-1.19)<br>1.00<br>1.02 (0.91-1.14)<br>1.29 (1.15-1.45)	Age, sex, practice, calendar time, COPD, cerebrovascular accident, diabetes, heart failure, hyperthyroidism, IHD, hypertension, bisphosphonates, oral glucocorticoids, statins, xanthine derivate, beta-2 agonists, drugs affecting the renin-angiotensin-aldosterone system, alcohol, smoking
Grundvold I et al, 2012, Norway	Oslo Cardiovascular Health Survey	1972-1975 – 2008, 30 years follow-up	2014 men, age 40-59 years: 270 AF cases	Measured	BMI	Per 2.8 units	1.16 (1.02-1.32)	Age, SBP, DBP, physical fitness, exercise maximal heart rated, pulse pressure, exercise maximal SBP
Korda RJ et al, 2012, Australia	The 45 and Up Study	2006-2009, 2.3 years follow-up	246361 men and women, age ≥45 years: 1880 AF cases	Self-reported	BMI, age 45-64 years<br><br>BMI, age 65-79 years<br><br>BMI, age ≥80 years	18.5-<25.0<br>25.0-<30.0<br>≥30.0<br>18.5-<25.0<br>25.0-<30.0<br>≥30.0<br>18.5-<25.0<br>25.0-<30.0<br>≥30.0	1.00<br>1.37 (1.09-1.72)<br>1.84 (1.45-2.33)<br>1.00<br>1.07 (0.91-1.26)<br>1.33 (1.11-1.60)<br>1.00<br>1.04 (0.87-1.25)<br>1.15 (0.88-1.50)	Age, sex, region of residence, household income, smoking, alcohol, private health insurance status,
Alonso A et al, 2013, USA	Framingham Heart Study Offspring cohort	1995-1998 - 2005, NA years of follow-up	2838 men and women, mean age 60 years: 143 cases	Measured	Weight	Per 15 kg	1.22 (1.03-1.44)	Age, sex
Alonso A et al, 2013, USA	Cardiovascular Health Study	1989-1990, 1992-1993 - 2000, NA years	5043 men and women, age ≥65 years: 624 cases	Measured	Weight, whites<br>Weight, blacks	Per 15 kg<br>Per 15 kg	1.18 (1.07-1.31)<br>1.31 (1.04-1.65)	Age, sex

		of follow-up						
Alonso A et al, 2013, USA	Atherosclerosis Risk in Communities Cohort	1996-1998, - 2005, NA years of follow-up	10675 men and women, age 45-64 years: 419 cases	Measured	Weight, whites<br>Weight, blacks	Per 15 kg<br>Per 15 kg	1.39 (1.26-1.53)<br>1.44 (1.08-1.91)	Age, sex
Alonso A et al, 2013, Island	Age, Gene/ Environment Susceptibility Reykjavik Study	2002-2006 - 2011, NA years of follow-up	4469 men and women, mean age 76 years: 408 cases	Measured	Weight	Per 15 kg	1.17 (1.04-1.31)	Age, sex
Alonso A et al, 2013, Netherlands	Rotterdam Study	1997-2005, NA years of follow-up	3203 men and women, age ≥55 years: 177 cases	Measured	Weight	Per 15 kg	1.55 (1.28-1.87)	Age, sex
Conen D et al, 2013, USA	Women's Health Study	NA-2011, 16.4 years follow-up	34713 women, age ≥45 years: 796 AF cases	Self-reported	Body weight	Per 10 kg	1.07 (0.998-1.14)	Age, hypertension, diabetes, race, education, alcohol, smoking, exercise, height
Karasoy D et al, 2013, Denmark	Danish Pregnant Women	2004-2009 - 2010, 4.6 years follow-up	271203 women, mean age 30.6 years: 110 AF hospitalizations	Measured	BMI	<18.5<br>18.5-25<br>25-29.9<br>30-35<br>>35	1.65 (0.88-3.35)<br>1.00<br>1.13 (0.68-1.88)<br>2.04 (1.13-3.69)<br>3.50 (1.86-6.58)	Age, hyperthyroidism, previous use of beta-blockers
Nyrnes A et al, 2013, Norway	The Tromsø study	1994-1995 - 2007, 11 years follow-up	22815 men and women, age 25-96 years: 461/361 AF cases	Measured	BMI, all AF, men<br>BMI, lone AF, men<br>BMI, all AF, women<br>BMI, lone AF, women	Per 3.2 units<br>Per 3.2 units<br>Per 4.1 units<br>Per 4.1 units	1.16 (1.06-1.27)<br>1.47 (1.32-1.63)<br>1.47 (0.90-2.40)<br>2.13 (1.47-3.09)	Age, height, total cholesterol, HDL-cholesterol, hypertension, palpitations, diabetes (all AF only), CHD (all AF only)
Azarbal F et al, 2014, USA	Women's Health Initiative	1994-1998 – 2011, 11.5 years follow-up	81317 women, age 50-79 years: 9792 AF cases	Measured	BMI	Per 5 units	1.12 (1.10-1.14)	Age, race/ethnicity, education, hypertension, diabetes, hyperlipidemia, CAD, heart failure, PAD, current smoking, physical activity
Reeves GK et al, 2014, United Kingdom	Million Women's Study	1996-2001 – 2008, 9.2 years follow-up	1251619 women, age 50-64 years: 11078 AF hospitalizations	Self-reported	BMI	<22.5<br>22.5-24.9<br>25.0-29.9<br>30.0-34.9<br>≥35.0<br>Per 5 units	0.95 (0.91-1.00)<br>1.00 (0.96-1.04)<br>1.24 (1.20-1.28)<br>1.75 (1.67-1.82)<br>2.90 (2.74-3.07)<br>1.43 (1.40-1.46)	Age, geographical region, SES, age at 1st birth, parity, smoking status, alcohol intake, physical activity, time since menopause, HRT use
Huxley RR et	Atherosclerosis	1987-2009,	14219 men and	Measured	BMI	18.5-24.9	1.00	Age, sex, race, study site, education,

al, 2014, USA	Risk in Communities Study	~18.2 years follow-up	women, age 45-64 years: 1775 AF cases		 <br>Waist circumference<br><br><br><br>Weight change, men<br><br><br><br><br>Weight change, women	25.0-29.9<br>≥30.0<br>≤92/≤84 cm<br>93-98/85-94<br>99-105/95-105<br>≥106/≥106<br>>5% loss<br>0-5% loss<br>0 to 4.9% gain<br>5-9.9 gain<br>≥10% gain<br>>5% loss<br>0-5% loss<br>0 to 4.9% gain<br>5-9.9 gain<br>≥10% gain	1.20 (1.06-1.36)<br>1.95 (1.72-2.21)<br>1.00<br>1.18 (1.01-1.37)<br>1.52 (1.31-1.76)<br>2.13 (1.85-2.46)<br>1.52 (1.16-1.99)<br>1.01 (0.79-1.31)<br>1.00<br>1.33 (1.04-1.70)<br>1.61 (1.24-2.11)<br>1.45 (1.08-1.93)<br>1.24 (0.93-1.64)<br>1.00<br>1.24 (0.94-1.62)<br>1.19 (0.90-1.55)	income, prior CVD, cigarette smoking, height, physical activity, alcohol
Sandhu RK et al, 2014, USA	Women's Health Study	1993 – 2004, 16.4 years follow-up	34720 women, age ≥45 years: 690 paroxysmal AF cases<br>349 nonparoxysmal AF cases	Self-reported	BMI, paroxysmal AF<br><br><br><br>BMI, nonparoxysmal AF<br><br><br><br>BMI, paroxysmal AF<br><br><br><br>BMI, nonparoxysmal AF	<25<br>25-<30<br>≥30<br>Per 1 unit<br><25<br>25-<30<br>≥30<br>Per 1 unit<br><25<br>25-<30<br>≥30<br>Per 1 unit<br><25<br>25-<30<br>≥30<br>Per 1 unit	1.00<br>1.30 (1.08-1.55)<br>1.49 (1.22-1.83)<br>1.03 (1.02-1.05)<br>1.00<br>1.51 (1.15-1.97)<br>2.56 (1.93-3.40)<br>1.07 (1.05-1.09)<br>1.00<br>1.30 (1.08-1.55)<br>1.50 (1.22-1.84)<br>1.03 (1.02-1.05)<br>1.00<br>1.51 (1.15-1.97)<br>2.57 (1.93-3.40)<br>1.07 (1.05-1.09)	Age, aspirin, vitamin E, beta-carotene, hypertension, diabetes, cholesterol, alcohol, smoking, exercise,<br><br><br><br><br>+ interim MI, stroke, revascularization, heart failure
Frost L et al, 2014, Denmark	Danish Diet, Cancer and Health Study	1993-1997 - 13.5 years follow-up	55273 men and women, age 50-64 years: 1669/912 AF cases	Measured	Weight<br>Weight, adj. height<br>BMI<br>Waist circumference<br>Hip circumference<br>WHR<br>Fat mass	Per 14.1 kg<br>Per 14.1 kg<br>Per 4.1 units<br>Per 12.7 cm<br>Per 7.9 cm<br>Per 0.10 units<br>Per 8.3 kg	1.36 (1.31-1.40)<br>1.29 (1.24-1.34)<br>1.26 (1.21-1.30)<br>1.28 (1.23-1.33)<br>1.29 (1.24-1.33)<br>1.11 (1.07-1.16)<br>1.29 (1.24-1.33)	Age, smoking status, fruit and vegetables, alcohol, physical activity, total energy, education, hypertension, diabetes, hypercholesterolemia, IHD, CHF, vascular heart disease

					Fat mass, adj. LBM<br>Fat%<br>Lean body mass<br>LBM, adj. height<br>LBM, adj. fat mass	Per 8.3 kg<br>Per 7.4%<br>Per 9.7 kg<br>Per 9.7 kg<br>Per 9.7 kg	1.03 (0.99-1.09)<br>1.19 (1.14-1.24)<br>1.40 (1.35-1.45)<br>1.38 (1.32-1.45)<br>1.37 (1.30-1.44)	
Schmidt M et al, 2014, Denmark	Danish Military Conscripts	1977-2012, 26 years follow-up	12850 men, median age 19 years: 227 AF cases	Measured	BMI	<18.5<br>18.5-24.9<br>25.0-29.9<br>≥30.0	0.99 (0.52-1.87)<br>1.00<br>2.08 (1.48-2.92)<br>2.87 (1.46-5.62)	Years of education, height
Knuiman M et al, 2014, Australia	The Busselton Health Study	1994-1995 - 2010, 15 years follow-up	4267 men and women, age 25-84 years: 343 AF cases	Measured	BMI<br>WC<br>WHR	Per 4.2 units<br>Per 12.7 cm<br>Per 0.09 units	1.34 (1.21-1.49)<br>1.37 (1.21-1.55)<br>1.21 (1.02-1.43)	Age, sex, height
Aronis KN et al, 2015, USA	Health, Aging, and Body Composition Study	1997-1998 – 2008, 10 years follow-up	2717 men and women, mean age 74 years: 371 AF cases	Measured	BMI, all<br>Abdominal circumference<br>SAT<br>VAT<br>SAT+VAT<br>Total fat mass<br>Total fat percent<br>BMI, whites<br>Abdominal circumference<br>SAT<br>VAT<br>SAT+VAT<br>Total fat mass<br>Total fat percent<br>BMI, blacks<br>Abdominal circumference<br>SAT<br>VAT<br>SAT+VAT<br>Total fat mass<br>Total fat percent	Per 4.7 units<br>Per 13 cm<br><br>Per 120 cm^2<br>Per 67 cm^2<br>Per 1 SD<br>Per 9 kg<br>Per 8%<br>Per 4.7 units<br>Per 13 cm<br><br>Per 120 cm^2<br>Per 67 cm^2<br>Per 1 SD<br>Per 9 kg<br>Per 8%<br>Per 4.7 units<br>Per 13 cm<br><br>Per 120 cm^2<br>Per 67 cm^2<br>Per 1 SD<br>Per 9 kg<br>Per 8%	1.14 (1.02-1.28)<br>1.16 (1.04-1.28)<br><br>1.11 (0.97-1.27)<br>1.07 (0.96-1.19)<br>1.10 (0.98-1.25)<br>1.13 (1.002-1.27)<br>1.03 (0.87-1.22)<br>1.11 (0.96-1.30)<br>1.13 (0.94-1.36)<br><br>1.16 (0.98-1.38)<br>1.04 (0.92-1.19)<br>1.12 (0.96-1.30)<br>1.16 (0.99-1.34)<br>1.06 (0.85-1.31)<br>1.13 (0.94-1.36)<br>1.27 (1.06-1.52)<br><br>0.98 (0.78-1.22)<br>1.16 (0.93-1.45)<br>1.04 (0.84-1.29)<br>1.04 (0.86-1.27)<br>0.92 (0.69-1.23)	Age, sex, race, site, smoking, adiposity measure, systolic and diastolic blood pressure, treatment of hypertension, total to HDL cholesterol ratio, heart rate, ECG left ventricular hypertrophy, PR interval, prevalent heart failure, coronary artery disease, diabetes
Azarbal F et al, 2015, USA	Women's Health Initiative	1994-1998 – 2011,11.6 years follow-up	8832 women, mean age 63.3 years: 1035 AF cases	Measured	BMI<br>Total body lean mass<br>Central lean mass	Per 5 units<br>Per 5 units<br>Per 5 units	1.07 (1.01-1.13)<br>1.21 (1.14-1.29)<br>1.49 (1.31-1.69)	Age, ethnicity, education, hypertension, diabetes, hyperlipidemia, coronary artery

					Peripheral lean mass<br>Total LBMI<br>Central LBMI<br>Peripheral LBMI<br>Total body fat mass<br>Central fat mass<br>Peripheral fat mass<br>Total body fat%<br>Central fat% of central mass<br>Central fat% of total fat mass	Per 5 units<br>Per 5 units<br>Per 5 units<br>Per 5 units<br>Per 5 kg<br>Per 5 kg<br>Per 5 kg<br>Per 5 %<br>Per 5 %<br><br>Per 5 %	1.37 (1.21-1.56)<br>1.39 (1.15-1.67)<br>2.03 (1.41-2.91)<br>1.74 (1.22-2.50)<br>1.04 (1.01-1.07)<br>1.05 (1.00-1.11)<br>1.10 (1.04-1.16)<br>1.00 (0.95-1.04)<br>0.98 (0.94-1.03)<br><br>0.95 (0.90-0.99)	disease, heart failure, peripheral artery disease, smoking, dietary modification, and hormone intervention
Vermond RA et al, 2015, Netherlands	Prevention of Renal and Vascular End-Stage Disease	NA-2008, 9.7 years follow-up	8265 men and women, age 28-75 years: 265 AF cases	Measured	BMI	Per 5 units	1.45 (1.21-1.74)	Age, sex
Nyström PK et al, 2015, Sweden	Stockholm County Study	1997-1999 – 2012, 13.6 years follow-up	1924 men and 2097 women, age 60 years: 285 AF cases	Measured	Weight<br>Waist circumference<br>Hip circumference<br>SAD<br>BMI<br>WHR<br>BMI, no MetS<br>BMI, MetS<br>BMI, no MetS<br>BMI, MetS<br>BMI, no MetS<br>BMI, MetS<br>WC, no MetS<br>WC, MetS<br>WC, no MetS<br>WC, MetS<br>WC, no MetS<br>WC, MetS	Per 14.2 kg<br>Per 12.4 cm<br>Per 8.4 cm<br>Per 2.9 cm<br>Per 4.2 kg/m^2<br>Per 0.087 units<br>18.5-24.9<br>18.5-24.9<br>25.0-29.9<br>25.0-29.9<br>≥30.0<br>≥30.0<br><94/<80 cm<br><94/<80 cm<br>94-101.9/80-87.9<br>94-101.9/80-87.9<br>≥102/≥88<br>≥102/≥88	1.47 (1.31-1.65)<br>1.35 (1.19-1.54)<br>1.38 (1.24-1.53)<br>1.28 (1.14-1.44)<br>1.25 (1.12-1.40)<br>1.05 (0.88-1.25)<br>1.00<br>1.17 (0.51-2.70)<br>1.01 (0.73-1.40)<br>1.67 (1.16-2.41)<br>1.75 (1.11-2.74)<br>1.92 (1.34-2.74)<br>1.00<br>1.45 (0.88-2.38)<br>1.19 (0.80-1.78)<br>1.33 (0.86-2.04)<br>1.37 (0.91-2.07)<br>2.03 (1.44-2.87)	Hypertension, elevated fasting glucose, sex, smoking, alcohol, history of MI, regular moderate-intensity physical activity, Swedish-born
Kokubo Y et al, 2015, Japan	The Suita Study	1989-1996/1996-1998/1992-2006 – 2013, 12.8 years	6906 men and women, age 30-84 years: 253 AF cases	Measured	BMI	<18.5<br>18.5-<25.0<br>≥25.0	1.02 (0.60-1.72)<br>1.00<br>1.35 (1.01-1.80)	Age, sex, blood pressure, smoking, drinking, hyperlipidemia, diabetes mellitus, impaired fasting glucose

		follow-up						
Berkovitch A et al, 2016, Israel	The Institute for Medical Screening of the Chaim Sheba Medical Center	2000 - NA, 6.4 years follow-up	18290 men and women, mean age 48.9 years: 288 AF cases	Measured	BMI	18.5-24.9<br>25.0-29.9<br>≥30.0	1.00<br>1.49 (1.11-2.00)<br>2.34 (1.64-3.34)	Age, sex, IHD, diabetes mellitus, hypertension, LDL cholesterol, HDL cholesterol, physical activity
Diouf I et al, 2016, Australia	Australian Diabetes, Obesity and Lifestyle study cohort	1999/2000 - 2004/2005, 5 years follow-up	5389 men and women, age ≥35 years: 53 AF cases	Measured	BMI	18.5-24.9<br>25.0-29.9<br>≥30.0	1.0<br>1.0 (0.7-1.5)<br>1.4 (0.5-4.2)	Age, sex, smoking status, usual number of alcoholic drinks, physical activity, level of education
Karas MG et al, 2016, USA	Cardiovascular Health Study	1989-2008, 13 years follow-up	4276 men and women, age ≥65 years: 1050 AF cases	Measured	BMI<br>Weight<br>Waist circumference<br>Hip circumference<br>WHR<br>Fat mass<br>Fat-free mass	Per 4.7 units<br>Per 14.6 kg<br>Per 13.2 cm<br>Per 10.0 cm<br>Per 0.09 units<br>Per 10.8 kg<br>Per 9.2 kg	1.08 (1.02-1.15)<br>1.21 (1.13-1.29)<br>1.14 (1.07-1.22)<br>1.17 (1.10-1.24)<br>1.02 (0.96-1.10)<br>1.16 (1.09-1.24)<br>1.26 (1.13-1.40)	Age, sex, race, smoking status
Kang SH et al, 2016, Korea	Korea National Health Insurance Corporation Study	2003-2004 - NA, 9.0 years follow-up	132063 men and women, age ≥40 years: 3237 AF cases	Measured	BMI, all<br><br><br><br><br>BMI, men<br><br><br><br><br>BMI, women	<18.5<br>18.5-22.9<br>23.0-24.9<br>25.0-29.9<br>≥30.0<br><18.5<br>18.5-22.9<br>23.0-24.9<br>25.0-29.9<br>≥30.0<br><18.5<br>18.5-22.9<br>23.0-24.9<br>25.0-29.9<br>≥30.0	1.23 (1.00-1.52)<br>1.00<br>1.15 (1.05-1.27)<br>1.26 (1.16-1.38)<br>2.20 (1.87-2.59)<br>1.17 (0.90-1.53)<br>1.00<br>1.09 (0.97-1.23)<br>1.22 (1.09-1.37)<br>2.17 (1.71-2.75)<br>1.32 (0.95-1.84)<br>1.00<br>1.24 (1.07-1.44)<br>1.30 (1.13-1.50)<br>2.22 (1.77-2.79)	Age, sex, alcohol, exercise frequency, hypertension, diabetes, ischemic heart disease, congestive heart failure, chronic kidney disease, chronic lung disease, malignancy, thyroid disease, fasting glucose, urine protein
Lee JJ et al, 2016, USA	Framingham Heart Study Offspring and Third-Generation	1998-2001 and 2002-2005 - NA, 9.7 years follow-up	2135 men and women, age : 162 AF cases	Measured	Pericardial fat<br>Intrathoracic fat<br>Abdominal visceral fat	Per 46 cm^3<br>Per 63 cm^3<br>Per 1061 cm^3	1.13 (0.99-1.30)<br>1.19 (1.01-1.40)<br>1.09 (0.93-1.28)	Age, sex, SBP, DBP, current smoking, antihypertensive medication use, diabetes mellitus, history of heart failure, history of myocardial infarction

	Cohorts							

AF=atrial fibrillation, BMI=body mass index, CHD=coronary heart disease, CHF=congestive heart failure, COPD=chronic obstructive pulmonary disease, CVD=cardiovascular disease, DBP=diastolic blood pressure, ECG=electrocardiogram, FEV_1=forced expiratory volume in 1 second, FH=family history, GFR=glomerular filtration rate, HDL=high density lipoprotein, HF=heart failure, HOMA-IR= homeostatic model assessment of insulin resistance, IHD=ischemic heart disease, LDL=low-density lipoprotein, LVH=left ventricular hypertrophy, MH=metabolically health, MI=myocardial infarction, MUH=metabolically unhealthy, NA=not available SBP=systolic blood pressure, WHI=Women's Health Initiative, WHR= waist-to-hip ratio

Supplementary Table 5. Table of RRs and 95% CIs from nonlinear dose-response analysis of BMI and atrial fibrillation

	Atrial fibrillation
BMI	RR (95% CI)
15.0	0.98 (0.90-1.06)
17.5	0.96 (0.93-1.00)
20.0	1.00
22.5	1.09 (1.04-1.13)
25.0	1.21 (1.13-1.31)
27.5	1.39 (1.25-1.56)
30.0	1.63 (1.40-1.89)
32.5	1.93 (1.60-2.32)
35.0	2.32 (1.86-2.90)
37.5	2.82 (2.18-3.66)
40.0	3.45 (2.56-4.64)

Supplementary Table 6. Table of RRs and 95% CIs from nonlinear dose-response analysis of waist circumference and atrial fibrillation

Men	
WC	RR (95% CI)
76 cm	1.00
80	1.01 (0.89-1.15)
85	1.07 (0.85-1.35)
90	1.18 (0.88-1.57)
95	1.34 (0.97-1.84)
100	1.55 (1.13-2.14)
105	1.84 (1.36-2.49)
110	2.22 (1.69-2.92)

Supplementary Table 7. Table of summary relative risks (95% CIs) from the current meta-analysis

Adiposity variable	N	Increment	Summary RR (95% CI)	I^2	$p_{heterogeneity}$
BMI	25	5 kg/m^2	1.28 (1.20-1.38)	97%	<0.0001
Waist circumference	5	10 cm	1.18 (1.12-1.25)	73%	0.005
Waist-to-hip ratio	4	0.1 units	1.09 (1.02-1.16)	44%	0.15
Hip circumference	3	10 cm	1.32 (1.16-1.51)	91%	<0.0001
Weight	10	5 kg	1.10 (1.08-1.13)	74%	<0.0001
Weight gain	2	5 kg	1.08 (0.97-1.19)	86%	0.007
Body fat mass	4	5 kg	1.09 (1.02-1.16)	94%	<0.0001
Body fat percentage	3	10%	1.10 (0.92-1.33)	90%	<0.0001

Supplementary Figure 1. Funnel plot for BMI and atrial fibrillation incidence

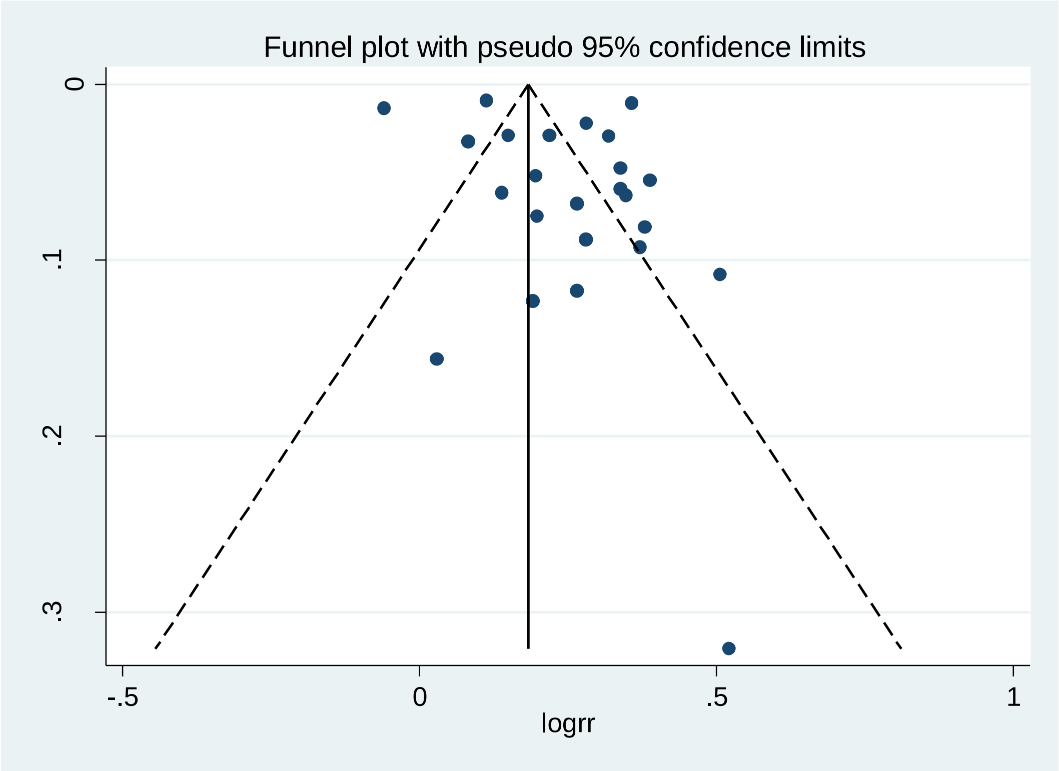

Moose checklist

Reporting of background should include	Page
Problem definition	3-4
Hypothesis statement	4
Description of study outcome(s)	3-4, 5
Type of exposure or intervention used	3-4, 5
Type of study designs used	3-4, 5
Study population	5, Supplementary Table 4
Reporting of search strategy should include	
Qualifications of searchers (eg, librarians and investigators)	5, Investigators
Search strategy, including time period included in the synthesis and keywords	Supplementary Table 1 and 2
Effort to include all available studies, including contact with authors	No contact with authors
Databases and registries searched	5
Search software used, name and version, including special features used (eg, explosion)	5, Supplementary Table 1 and 2
Use of hand searching (eg, reference lists of obtained articles)	5
List of citations located and those excluded, including justification	Supplementary Table 3
Method of addressing articles published in languages other than English	5, only English language publications were included
Method of handling abstracts and unpublished studies	5, not included
Description of any contact with authors	No contact with authors
Reporting of methods should include	
Description of relevance or appropriateness of studies assembled for assessing the hypothesis to be tested	5-6
Rationale for the selection and coding of data (eg, sound clinical principles or convenience)	5-6
Documentation of how data were classified and coded (eg, multiple raters, blinding, and interrater reliability)	6
Assessment of confounding (eg, comparability of cases and controls in studies where appropriate)	Table 1, Supplementary Table 4
Assessment of study quality, including blinding of quality assessors; stratification or regression on possible predictors of study results	5, 11, Table 1
Assessment of heterogeneity	7

Description of statistical methods (eg, complete description of fixed or random effects models, justification of whether the chosen models account for predictors of study results, dose-response models, or cumulative meta-analysis) in sufficient detail to be replicated	6-7
Provision of appropriate tables and graphics	8-11, Supplementary Table 1-4
Reporting of results should include	
Graphic summarizing individual study estimates and overall estimate	8-11, Figure 2-5, Table 1
Table giving descriptive information for each study included	Supplementary Table 4
Results of sensitivity testing (eg, subgroup analysis)	10-11, Table 1
Indication of statistical uncertainty of findings	8-11
Reporting of discussion should include	
Quantitative assessment of bias (eg, publication bias)	14
Justification for exclusion (eg, exclusion of non–English-language citations)	No relevant non-English publications were identified
Assessment of quality of included studies	15
Reporting of conclusions should include	
Consideration of alternative explanations for observed results	13-14
Generalization of the conclusions (ie, appropriate for the data presented and within the domain of the literature review)	14-15
Guidelines for future research	15
Disclosure of funding source	16
